# Deletion of the murine ortholog of human 9p21.3 locus promotes atherosclerosis by increasing macrophage proinflammatory activity

**DOI:** 10.3389/fcvm.2023.1113890

**Published:** 2023-03-06

**Authors:** Sanna Kettunen, Anna-Kaisa Ruotsalainen, Tiit Örd, Tuisku Suoranta, Janne Heikkilä, Minna U. Kaikkonen, Nihay Laham-Karam, Seppo Ylä-Herttuala

**Affiliations:** ^1^A.I. Virtanen Institute, University of Eastern Finland, Kuopio, Finland; ^2^Cancer Center, Kuopio University Hospital, Kuopio, Finland; ^3^Heart Center and Gene Therapy Unit, Kuopio University Hospital, Kuopio, Finland

**Keywords:** *ANRIL*, atherosclerosis, inflammation, coronary artery disease, Chr9p21.3, macrophage, mouse model

## Abstract

**Background:**

Several genome-wide association studies have reported a risk locus for coronary artery disease (CAD) in the 9p21. 3 chromosomal region. This region encodes a lncRNA in the INK4 locus (*ANRIL*) and its genetic variance has a strong association with CAD, but its mechanisms in atherogenesis remain unclear.

**Objectives:**

This study aimed to investigate the role of the murine ortholog of human 9p21.3 locus in atherogenesis in hypercholesterolemic mice.

**Methods:**

Murine 9p21.3 ortholog knockout mice (Chr4^Δ70*kb*/Δ70*kb*^) were crossbred with *Ldlr*^−/−^*ApoB*^100/100^ mice, and atherosclerotic plaque size and morphology were analyzed on a standard or a high-fat diet (HFD). The hematopoietic cell-specific effect of Chr4^Δ70*kb*/Δ70*kb*^ on atherosclerotic plaque development was studied *via* bone marrow (BM) transplantation, where Chr4^Δ70*kb*/Δ70*kb*^ or wild-type BM was transplanted into *Ldlr*^−/−^*ApoB*^100/100^ mice. The role of Chr4^Δ70*kb*/Δ70*kb*^ in macrophage M1/M2 polarization was studied. In addition, single-cell sequencing data from human and mouse atheroma were analyzed to show the expression profiles of *ANRIL* and its murine equivalent, *Ak148321*, in the plaques.

**Results:**

Both systemic and hematopoietic Chr4^Δ70*kb*/Δ70*kb*^ increased atherosclerosis in *Ldlr*^−/−^*ApoB*^100/100^ mice after 12 weeks of HFD. The systemic Chr4^Δ70*kb*/Δ70*kb*^ also elevated the number of circulating leukocytes. Chr4^Δ70*kb*/Δ70*kb*^ BMDMs showed enhanced M1 polarization *in vitro*. Single-cell sequencing data from human and mouse atheroma revealed that *ANRIL* and *Ak148321* were mainly expressed in the immune cells.

**Conclusion:**

These data demonstrate that both systemic and BM-specific deletion of the murine 9p21.3 risk locus ortholog promotes atherosclerosis and regulates macrophage pro-inflammatory activity, suggesting the inflammation-driven mechanisms of the risk locus on atherogenesis.

## 1. Introduction

Atherosclerosis is a progressive chronic inflammatory disease leading to the accumulation of cholesterol-containing plasma lipoproteins and inflammatory cells in the vascular wall of medium- and large-size arteries, narrowing the arterial lumen, impairing the blood flow to the tissue, and predisposing to severe cardiovascular complications, such as myocardial infarction (MI) and chronic heart failure (1). Intimal macrophages engulf cholesterol-rich low-density lipoprotein (LDL) particles, subsequently transform into foam cells, and form fatty streaks in the arterial wall. Macrophages express several pro-inflammatory cytokines and chemokines, attracting circulating monocytes and other inflammatory cells to enter the vascular wall (2). In addition, hyperlipidemia is associated with an increased number of circulating leukocytes (3). In the arterial wall, the polarization of tissue macrophages into pro-inflammatory M1 or anti-inflammatory M2 macrophages is critical in maintaining the local vascular repair processes in response to arterial lipid accumulation and thus contributing to the atherogenic processes (2).

Recent studies have suggested that several lncRNAs play a role in vascular biology in the regulation of atherogenesis and lipid metabolism (4). However, many of these lncRNAs and their functions in cardiovascular diseases have remained poorly characterized. In 2007, several single-nucleotide polymorphisms (SNPs) with the strongest association with coronary artery disease (CAD) were found in the short arm of human chromosome 9 (Chr9p21.3), within the region encoding an lncRNA in the *INK4* locus (*ANRIL*) (5). Interestingly, the CAD risk associated with this genomic locus has been proposed to be independent of conventional atherosclerosis risk factors, such as hyperlipidemia. *ANRIL* has multiple splice variants and both linear and circular forms that may have different impacts on atherosclerosis. It has been suggested that circular isoforms are protective against CAD, while linear forms have been considered pro-atherogenic (6). Several mechanisms by which *ANRIL* regulates atherogenesis have been proposed, but they still require further clarification. Circular *ANRIL* has been reported to provide atheroprotection by binding NOP14 and PES1 proteins, leading to the modulation of ribosomal RNA maturation and cellular functions such as proliferation and apoptosis (6). In human endothelial cells, *ANRIL* was reported to act as a component of the NF-κB pathway and regulate inflammatory genes downstream of *TNF* through direct binding with Yin Yang 1 (*YY1*) (7). In addition, *ANRIL* was suggested to regulate the phenotypic alteration and proliferation of vascular smooth muscle cells (SMCs) by affecting NADPH oxidase 1 (*NOX1*) activation *via* epigenetic regulation (8). Although no mouse model exists that fully recapitulates the human risk allele, two independent studies investigated the effects of deleting the murine orthologous region to the 9p21.3 risk locus on atherosclerosis. In a hypercholesterolemic *ApoE*^−/−^ background, the deletion increased the development and calcification of advanced atherosclerotic plaques (9) but had no effect on atherosclerosis in the wild-type (WT) background (10). Indeed, these studies give support to similar functions of *ANRIL* and its murine equivalent in vascular cells and atherogenesis in both human and mouse models, but further studies are required.

In this study, we aimed to clarify the systemic and hematopoietic cell-specific effects of the CAD risk locus both on early and advanced atherosclerosis by using a mouse model having the Chr4^Δ70*kb*/Δ70*kb*^ deletion orthologous to human CAD risk locus, including the middle exons of the murine *ANRIL* equivalent, *Ak148321*, in a more human-like atherosclerotic mouse model. We did this by cross-breeding Chr4^Δ70*kb*/Δ70*kb*^ mice with the *Ldlr*^−/−^*ApoB*^100/100^ mouse model, which has a more human-like plasma lipoprotein profile than *ApoE*^−/−^ mice used in previous studies (11). *ApoE*^−/−^ mice have lipoproteins mainly consisting of VLDL and chylomicron remnants, whereas in *Ldlr*^−/−^*ApoB*^100/100^ mice, the LDL fraction is dominant, and they do not express ApoB48. Moreover, ApoE has direct effects on macrophage- and T-lymphocyte function, in addition to immune responses in the vascular wall (12, 13). This study demonstrates that systemic Chr4^Δ70*kb*/Δ70*kb*^ promotes the development of atherosclerosis in *Ldlr*^−/−^*ApoB*^100/100^ mice in an HFD-dependent manner, with an increase in circulating leukocyte number. The pro-atherogenic mechanism in the hypercholesterolemic condition is possibly mediated *via* inflammatory cell-specific effects, as hematopoietic Chr4^Δ70*kb*/Δ70*kb*^ also promotes the development of advanced atherosclerotic plaques in *Ldlr*^−/−^*ApoB*^100/100^ mice, and enhanced macrophage pro-inflammatory activity in bone marrow-derived macrophages has been detected in response to oxLDL and IFN-γ. Despite the structural differences between human *ANRIL* and murine *Ak148321*, the data demonstrate similar atherosclerotic phenotypes both in Chr4^Δ70*kb*/Δ70*kb*^ mice and humans carrying the CAD risk SNPs.

## 2. Materials and methods

### 2.1. Methods summary

**Figure 1:**
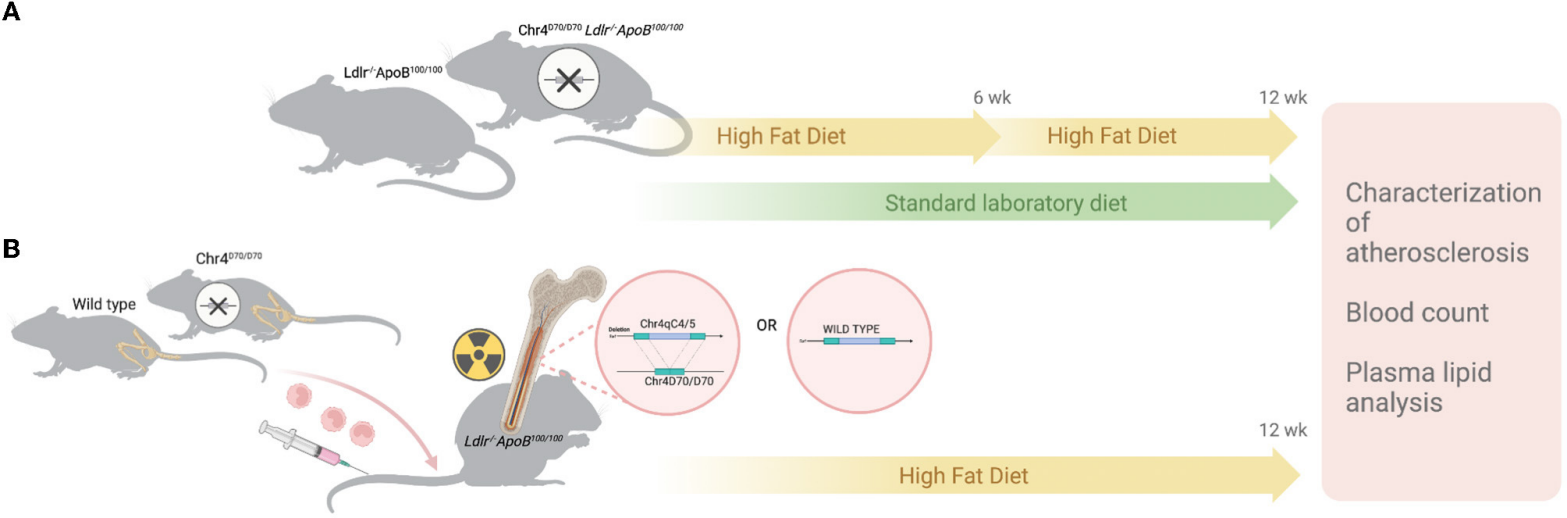
*In vivo* study protocol. Atherogenic status of hyperlipidemic *Ldlr*^−/−^*ApoB*^100/100^ mice with systemic and hematopoietic knockouts of the murine ortholog of the human 9p21.3 CAD risk interval was characterized on a standard laboratory diet and high fat diet (HFD). **(A)** Chr4^Δ70*kb*/Δ70*kb*^ *Ldlr*^−/−^*ApoB*^100/100^ and their littermate *Ldlr*^−/−^*ApoB*^100/100^ mice were fed a high-fat diet for 6 weeks (*n* = 9 + 10) or 12 weeks (*n* = 12 + 12) or with a standard laboratory diet for 12 weeks (*n* = 6 + 8). **(B)** *Ldlr*^−/−^*ApoB*^100/100^ mice with and without hematopoietic deficiency of human 9p21.3 CAD risk ortholog induced by Chr4^Δ70*kb*/Δ70*kb*^ (*n* = 19) or wild-type (*n* = 11) BM transplantation were a high-fat diet for 12 weeks. At the end of all study settings, mice were weighed, sacrificed, and their blood and tissues collected. The atherosclerosis status of the mice was characterized, and the blood glucose levels, plasma lipid levels, and white blood cell differential were measured.

To study the role of the murine ortholog of the human CAD risk interval in atherosclerosis, Chr4^Δ70*kb*/Δ70*kb*^ mice were backcrossed into the *Ldlr*^−/−^*ApoB*^100/100^ atherosclerotic mouse strain and studied on those fed the standard laboratory diet and on those fed HFD for 6 and 12 weeks (Figure 1A). The hematopoietic deletion was made by destroying the bone marrow (BM) of *Ldlr*^−/−^*ApoB*^100/100^ mice by irradiation and then transplanting new BM collected from Chr4^Δ70*kb*/Δ70*kb*^ or WT mice, after which the mice were fed HFD for 12 weeks (Figure 1B). The size and composition of atherosclerotic plaques were characterized by the histology and immunohistology of aortic roots. Plasma lipids and white blood cells were analyzed at the end of the study. To investigate the expression pattern of human *ANRIL* and its murine equivalent, *Ak148321*, in atherosclerotic plaques, scRNA-seq data generated by Wirka et al. (14) from human coronary arteries were used. For mouse atherosclerosis scRNA-seq, the raw sequencing reads generated by Pan et al. (15) from mouse aortas were obtained and reprocessed. Expression of CAD risk area transcripts in mouse spleen and the genes regulating lipid metabolism in the liver were measured with qPCR, using TaqMan-based assays. Mouse BMDMs were extracted, the cells were exposed to oxLDL for 16 h and the expression of pro-inflammatory cytokines was measured. M1/M2 phenotype polarization assay was performed, and foam cell formation and cell proliferation rate were measured in BMDMs. All animal experiments were approved by the National Experimental Animal Board of Finland and carried out following the guidelines of the Finnish Act on Animal Experimentation and Directive 2010/63/EU of the European Parliament. For statistical analyses, the Student's *t*-test or nonparametric Mann–Whitney test was used for testing the difference of means between the groups. The difference was considered statistically significant when the *P*-value was ≤ 0.05.

Detailed study methods are available in Supplementary material. All relevant data considered in this article are provided in Section 3 and Supplementary material, and raw data are available upon a reasonable request from the corresponding author.

## 3. Results

### 3.1. Systemic Chr4^Δ70*kb*/Δ70*kb*^ knockout increases atherosclerotic plaque development in *Ldlr^−/−^ApoB^100/100^* mice in an HFD-dependent manner

**Figure 2:**
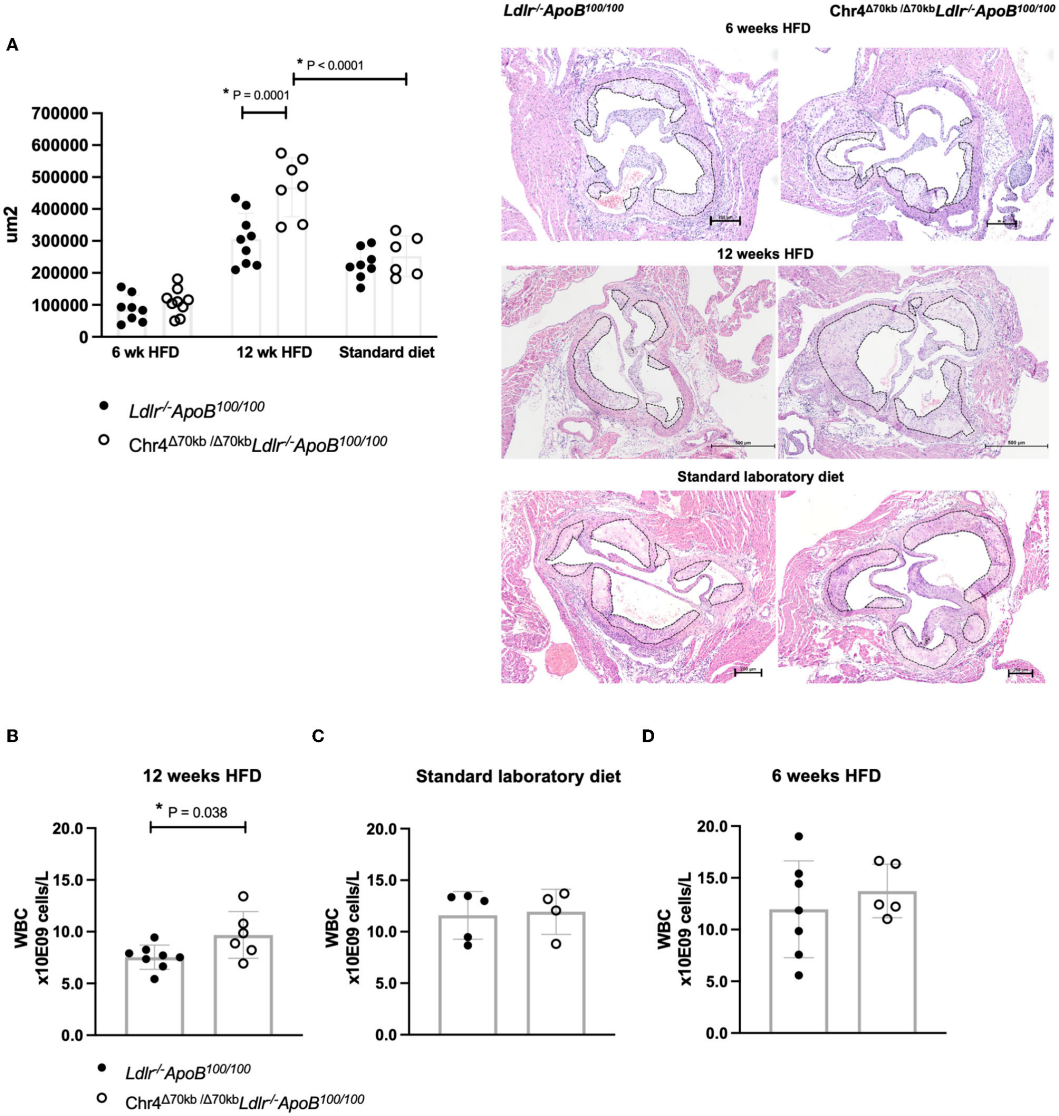
Systemic deletion of the murine ortholog of 9p21.3 risk locus promotes advanced atherosclerosis and increases the blood leukocytes in *Ldlr*^−/−^*ApoB*^100/100^ mice after 12 weeks of HFD. **(A)** Atherosclerotic plaque area in hematoxylin-eosin-stained cross sections of Chr4^Δ70*kb*/Δ70*kb*^ *Ldlr*^−/−^*ApoB*^100/100^ and *Ldlr*^−/−^*ApoB*^100/100^ mice aortic roots after 6 weeks of high-fat diet (HFD) (*n* = 9 + 8), as well as age-matched groups after 12 weeks of HFD (*n* = 7 + 9) and after standard laboratory diet (*n* = 6 + 8). **(B)** White blood cell count of Chr4^Δ70*kb*/Δ70*kb*^ *Ldlr*^−/−^*ApoB*^100/100^ (*n* = 6) and *Ldlr*^−/−^*ApoB*^100/100^ (*n* = 8) mice after 12 weeks of HFD, **(C)** after age-matched standard laboratory diet (*n* = 5 + 7), and **(D)** after 6 weeks of HFD (*n* = 4 + 5). The plaque area is illustrated with a dashed line in representative histology images. Graphs show mean ± SD. The data in graphs **(A, B, D)** were normally distributed and equal in variance. Analysis for statistically significant differences between and within the age-matched 12-week HFD and standard laboratory diet groups in graph **(A)** was performed by using *ANOVA* with Tukey's multiple comparison tests. Statistical analysis for graphs **(B, D)** was performed using the Student's *t*-test. Statistical analysis for graph **(C)** was performed using the Mann–Whitney test due to significant differences in variances. *P* ≤ 0.05 was considered a threshold for statistical significance.

To study the role of the murine equivalent of the CAD risk locus and *ANRIL* in atherosclerosis, we assessed the development of atherosclerotic plaques in hypercholesterolemic *Ldlr*^−/−^*ApoB*^100/100^ mice having a systemic deletion orthologous to the human CAD risk interval in Chr9p21.3. These Chr4^Δ70*kb*/Δ70*kb*^*Ldlr*^−/−^*ApoB*^100/100^ mice were fed HFD for 6 weeks to induce early atherosclerosis or aged until 6 months of age on standard diet or with 12 HFD for advanced plaques, respectively (Figure 1A). Chr4^Δ70*kb*/Δ70*kb*^ increased the development of advanced atherosclerotic plaques in an HFD-dependent manner (*P* = 0.0001; Figure 2A) in *Ldlr*^−/−^*ApoB*^100/100^ mice after 12 weeks of HFD, whereas there was no effect on atherosclerosis in age-matched mice on standard laboratory diet (Figure 2A). Interestingly, compared with standard diet, HFD increased the atherosclerotic plaque area in Chr4^Δ70*kb*/Δ70*kb*^*Ldlr*^−/−^*ApoB*^100/100^ mice almost two-fold (*P* < 0.0001) but did not significantly affect plaque size in *Ldlr*^−/−^*ApoB*^100/100^ control mice (*P* = 0.144). In addition, Chr4^Δ70*kb*/Δ70*kb*^ did not have an effect on early atherosclerosis that was measured after 6 weeks of HFD (Figure 2A). The effect of Chr4^Δ70*kb*/Δ70*kb*^ on atherosclerotic plaque morphology was analyzed, but there were no statistically significant differences in the total amount of macrophages, collagen, necrosis, or SMCs (data not shown) or the percentages of these characteristics in relation to the plaque area on the standard laboratory diet (Supplementary Figures 1A–D) or after 6 (Supplementary Figures 2A–E) or 12 weeks of HFD (Supplementary Figures 3A–E). Thus, HFD triggered an increase in advanced plaque area in Chr4^Δ70*kb*/Δ70*kb*^*Ldlr*^−/−^*ApoB*^100/100^ mice compared with *Ldlr*^−/−^*ApoB*^100/100^, which was caused by a gradual increase in multiple plaque characteristics. Plaque lymphocytes were analyzed after 6 (Supplementary Figure 2B) and 12 weeks of HFD (Supplementary Figure 3B), but their number was low, leaving macrophages as the main inflammatory cell type in the plaques.

Generally, elevated plasma total cholesterol and lipoprotein levels due to HFD are known to increase the number of circulating leukocytes (16, 17). Interestingly, the total number of blood leukocytes in Chr4^Δ70*kb*/Δ70*kb*^*Ldlr*^−/−^*ApoB*^100/100^ mice was increased (9.68 ± 2.26 × 10^9^ cells/L) compared with *Ldlr*^−/−^*ApoB*^100/100^ mice (7.53 ± 1.17 × 10^9^ cells/L; *P* = 0.038; Figure 2B) after 12 weeks of HFD, but Chr4^Δ70*kb*/Δ70*kb*^ did not affect the total number of blood leukocytes on the standard laboratory diet (Figure 2C) or after 6 weeks of HFD (Figure 2D). There was no difference in white blood cell differential count between the genotypes on a standard laboratory diet or after 6 or 12 weeks of HFD (Table 1). Body weight, plasma total cholesterol, LDL, HDL, and triglyceride levels were equal in Chr4^Δ70*kb*/Δ70*kb*^*Ldlr*^−/−^*ApoB*^100/100^ and *Ldlr*^−/−^*ApoB*^100/100^ mice on the standard laboratory diet and after 6 and 12 weeks of HFD (Table 1). Fasting blood glucose levels were higher in Chr4^Δ70*kb*/Δ70*kb*^*Ldlr*^−/−^*ApoB*^100/100^ mice (7.35 ± 0.58 mmol/L) compared with *Ldlr*^−/−^*ApoB*^100/100^ mice (6.59 ± 0.62 mmol/L) on the standard laboratory diet (*P* = 0.043; Table 1), but not in 6- or 12-week HFD mice.

**Table 1:** Characteristics of *Ldlr*^−/−^*ApoB*^100/100^ and Chr4^Δ70kb/Δ70kb^*Ldlr*^−/−^*ApoB*^100/100^ mice on standard and high fat diet.

	**12 weeks HFD**		**6 weeks HFD**		**Standard laboratory diet**	
**Variables**	***Ldlr** ^−/−^ **ApoB** ^100/100^*	**Chr4**^Δ70*kb*/Δ70*kb*^ ***Ldlr**^−/−^**ApoB**^100/100^*	***Ldlr** ^−/−^ **ApoB** ^100/100^*	**Chr4**^Δ70*kb*/Δ70*kb*^ ***Ldlr**^−/−^**ApoB**^100/100^*	***Ldlr** ^−/−^ **ApoB** ^100/100^*	**Chr4**^Δ70*kb*/Δ70*kb*^ ***Ldlr**^−/−^**ApoB**^100/100^*
Weight (g)	27.78 ± 4.81 (*n* = 12)	26.61 ± 4.49 (*n* = 12)	23.64 ± 1.83 (*n* = 10)	24.03 ± 3.93 (*n* = 9)	26.28 ± 4.25 (*n* = 8)	27.48 ± 5.87 (*n* = 6)
Weight gain (%)	38.79 ± 20.31 (*n* = 12)	33.56 ± 12.22 (*n* = 12)	15.15 ± 6.04 (*n* = 10)	15.52 ± 7.97 (*n* = 9)	16.46 ± 6.28 (*n* = 8)	25.37 ± 18.42 (*n* = 6)
Glucose (mmol/l)	6.86 ± 1.20 (*n* = 7)	6.46 ± 1.44 (*n* = 5)	7.41 ± 1.14 (*n* = 9)	7.37 ± 0.70 (*n* = 8)	6.59 ± 0.62 (*n* = 7)	7.35 ± 0.58 (*n* = 6) ^*^P **=** **0.046**
Total cholesterol (mmol/l)	22.09 ± 4.43 (*n* = 6)	22.73 ± 5.17 (*n* = 5)	22.85 ± 4.24 (*n* = 5)	20.07 ± 1.14 (*n* = 4)	7.47 ± 1.60 (*n* = 8)	6.82 ± 2.38 (*n* = 6)
LDL (mmol/l)	20.49 ± 4.27 (*n* = 6)	21.22 ± 3.21 (*n* = 5)	21.75 ± 4.38 (*n* = 5)	18.74 ± 1.00 (*n* = 4)	7.49 ± 1.66 (*n* = 8)	6.37 ± 2.01 (*n* = 6)
HDL (mmol/l)	8.98 ± 2.34 (*n* = 6)	7.27 ± 2.59 (*n* = 5)	10.83 ± 1.57 (*n* = 5)	8.84 ± 1.17 (*n* = 4)	3.61 ± 1.00 (*n* = 8)	3.19 ± 0.81 (*n* = 6)
Triglycerides (mmol/l)	1.46 ± 0.72 (*n* = 6)	1.33 ± 0.19 (*n* = 5)	1.46 ± 0.45 (*n* = 5)	1.31 ± 0.25 (*n* = 4)	1.37 ± 0.52 (*n* = 8)	1.18 ± 0.40 (*n* = 6)
%NEUT (%)	11.92 ± 4.49 (*n* = 6)	13.08 ± 4.99 (*n* = 5)	12.32 ± 2.60 (*n* = 5)	11.45 ± 1.72 (*n* = 4)	8.37 ± 1.46 (*n* = 7)	8.56 ± 2.14 (*n* = 5)
%LYM (%)	78.58 ± 5.78 (*n* = 6)	74.10 ± 4.10 (*n* = 5)	75.74 ± 6.15 (*n* = 5)	78.78 ± 4.24 (*n* = 4)	78.87 ± 3.78 (*n* = 7)	80.52 ± 6.35 (*n* = 5)
%MONO (%)	5.2 ± 1.25 (*n* = 6)	5.04 ± 0.85 (*n* = 5)	6.38 ± 1.63 (*n* = 5)	4.83 ± 1.93 (*n* = 4)	1.53 ± 0.25 (*n* = 7)	2.02 ± 0.85 (*n* = 5)
%EOS (%)	1.88 ± 0.73 (*n* = 6)	1.98 ± 0.89 (*n* = 5)	2.92 ± 1.52 (*n* = 5)	2.55 ± 0.56 (*n* = 4)	4.6 ± 1.64 (*n* = 7)	2.52 ± 0.76 (*n* = 5) ^*^P **=** **0.026**
%LUC (%)	2.05 ± 1.24 (*n* = 6)	2.73 ± 1.25 (*n* = 5)	2.34 ± 1.44 (*n* = 5)	2.15 ± 0.81 (*n* = 4)	6.27 ± 3.80 (*n* = 7)	5.72 ± 7.56 (*n* = 5)
%BASO (%)	0.33 ± 0.24 (*n* = 6)	0.78 ± 0.74 (*n* = 5)	0.26 ± 0.11 (*n* = 5)	0.25 ± 0.10 (*n* = 4)	0.39 ± 0.11 (*n* = 7)	0.72 ± 0.84 (*n* = 5)

Data are shown as mean ± SD. Statistical analyses were performed between Chr4^Δ70*kb*/Δ70*kb*^*Ldlr*^−/−^*ApoB*^100/100^ and *Ldlr*^−/−^*ApoB*^100/100^ mice within each diet group using a Student's *t*-test. The difference between Chr4^Δ70*kb*/Δ70*kb*^*Ldlr*^−/−^*Apo*^*B*100/100^ and *Ldlr*^−/−^*ApoB*^100/100^ groups was considered statistically significant when ^*^P ≤ 0.05. Significant *P* values are provided in the table.

### 3.2. Hematopoietic Chr4^Δ70*kb*/Δ70*kb*^ knockout promotes atherosclerosis in *Ldlr^−/−^ApoB^100/100^* mice

**Figure 3:**
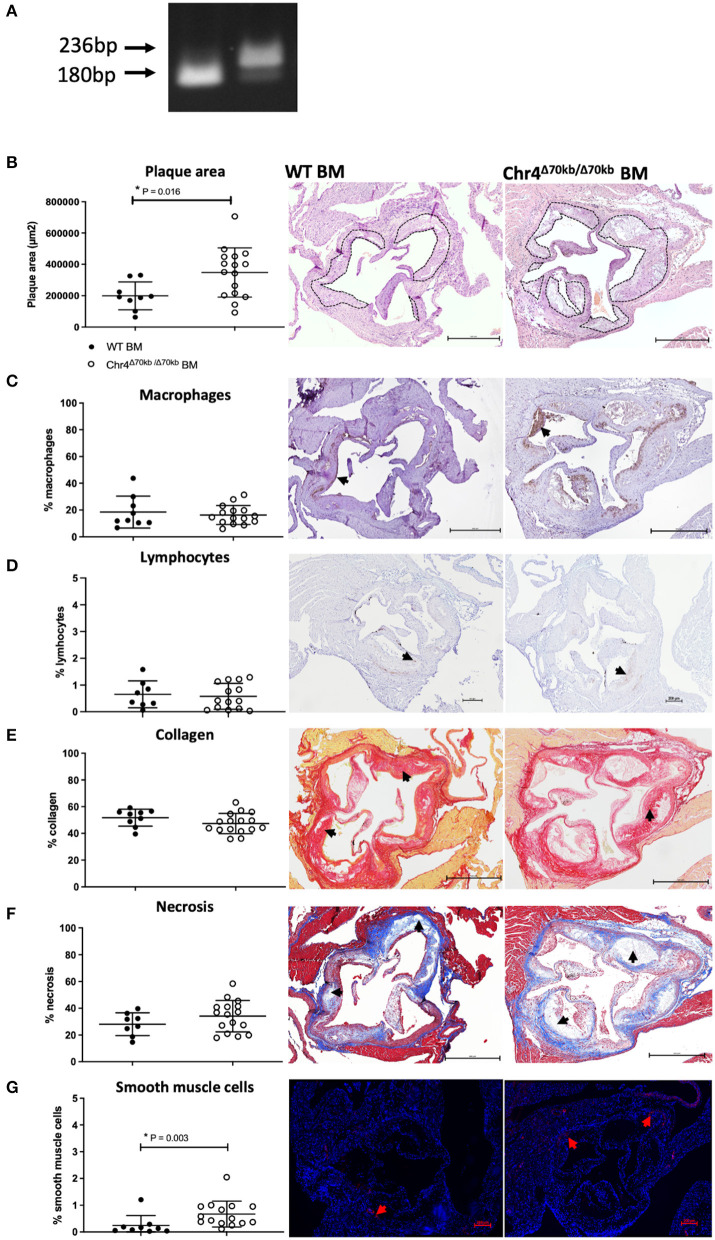
Hematopoietic Chr4^Δ70*kb*/Δ70*kb*^ increases the atherosclerotic plaque size of *Ldlr*^−/−^*ApoB*^100/100^ mice after 12 weeks of HFD. **(A)** Representative gel electrophoresis of white blood cell DNA after Chr4^Δ70*kb*/Δ70*kb*^ and wild-type (WT) bone marrow (BM) transplantation. WT is indicated by a 180 bp band, and Chr4^Δ70*kb*/Δ70*kb*^ is indicated by a 236 bp band. **(B)** Atherosclerotic plaque area of Chr4^Δ70*kb*/Δ70*kb*^ BM recipient (*n* = 16) and WT BM recipient (*n* = 10) *Ldlr*^−/−^*ApoB*^100/100^ mice in hematoxylin-eosin-stained cross-sections of the aortic root. The brown staining area represents the plaque macrophages, as demonstrated by arrowheads. **(C)** MAC3-positive % plaque area representing macrophages (*n* = 15 + 10). Arrowheads demonstrate positive staining. **(D)** CD3e-positive % plaque area representing lymphocytes (*n* = 8 + 14). Arrowheads demonstrate positive staining. **(E)** Sirius Red stained % plaque area (*n* = 16 + 10). The red staining area represents the plaque collagen, as demonstrated by arrowheads. **(F)** Plaque necrotic area % in Masson Trichrome staining (*n* = 16 + 9). A clear area on the plaques represents necrosis, as demonstrated by arrowheads. **(G)** Fluorescent aSMA-positive % area for plaque smooth muscle cells with DAPI counterstain for nucleus (*n* = 15 + 9). The red staining area represents the smooth muscle cells, as demonstrated by arrowheads. Graphs show mean ± SD. The data in graphs **(B–E)** were normally distributed and equal in variance, and analysis for statistical significance was performed using the Student's *t*-test. Statistical analysis for graph **(F)** was performed using the Mann–Whitney test due to its non-normal distribution and significant differences in variances. *P* ≤ 0.05 was considered a threshold for statistical significance.

As the Chr4^Δ70*kb*/Δ70*kb*^*Ldlr*^−/−^*ApoB*^100/100^ mice showed increased atherosclerosis and an elevated number of circulating leukocytes but no difference in plasma cholesterol levels after prolonged HFD, we investigated the effects of hematopoietic deficiency of the risk interval on atherosclerosis (Figure 1B). Therefore, WT or Chr4^Δ70*kb*/Δ70*kb*^ BM was transplanted into *Ldlr*^−/−^*ApoB*^100/100^ mice, and the engraftment of the transplant was determined from white blood cells (Figure 3A). We found that Chr4^Δ70*kb*/Δ70*kb*^ in BM-derived cells significantly increases the size of aortic root atherosclerotic plaques after 12 weeks of HFD in *Ldlr*^−/−^*ApoB*^100/100^ mice (*P* = 0.009; Figure 3B) compared with *Ldlr*^−/−^*ApoB*^100/100^ mice that received the WT BM. Characterization of plaque morphology revealed no statistically significant differences in total area (data not shown) and proportion of macrophages (Figure 3C), lymphocytes (Figure 3D), collagen (Figure 3E), or necrosis (Figure 3F) in relation to the plaque area between the BM transplant groups. Hematopoietic Chr4^Δ70*kb*/Δ70*kb*^ increased the area of SMCs with respect to the total plaque area compared with *Ldlr*^−/−^*ApoB*^100/100^ mice with WT BM (*P* = 0.004; Figure 3G). However, the number of SMCs was low, averaging < 1% of the plaque area in both groups. Parallel to total knockout, Chr4^Δ70*kb*/Δ70*kb*^ BM-transplanted mice also showed a tendency to increase blood leukocyte number (12.09 ± 3.65 × 10^9^ cells/L) compared with WT BM recipients (9.42 ± 2.82 × 10^9^ cells/L), but the difference between the groups did not reach statistical significance (*P* = 0.087; Table 2). The white blood cell differential counts were similar between the groups.

**Table 2:** Characteristics of *Ldlr*^−/−^*ApoB*^100/100^ mice after Chr4^Δ70kb/Δ70kb^ or wild type bone marrow transplantation and 12 weeks high fat diet.

	**Bone marrow transplant**		
	**WT**	**Chr4** ^Δ70*kb*/Δ70*kb*^	***p***
Weight (g)	27.19 ± 4.59 (*n* = 9)	29.10 ± 2.85 (*n* = 17)	0.201
Weight gain (%)	14.27 ± 13.94 (*n* = 9)	27.24 ± 10.07 (*n* = 17)	^*^ **0.012**
Glucose (mmol/l)	7.44 ± 1.09 (*n* = 9)	8.25 ± 0.93 (*n* = 17)	0.054
Total cholesterol (mmol/l)	22.35 ± 3.03 (*n* = 9)	26.91 ± 3.77 (*n* = 9)	^*^ **0.019**
LDL (mmol/l)	20.36 ± 2.29 (*n* = 9)	22.17 ± 3.25 (*n* = 9)	0.189
HDL (mmol/l)	7.12 ± 1.75 (*n* = 9)	7.86 ± 1.16 (*n* = 9)	0.304
Triglycerides (mmol/l)	1.60 ± 0.57 (*n* = 9)	3.31 ± 1.03 (*n* = 9)	^*^ **0.001**
WBCB (x10E09 cells/L)	9.42 ± 2.82 (*n* = 8)	12.09 ± 3.65 (*n* = 15)	0.087
RBC (x10E12 cells/L)	9.47 ± 0.79 (*n* = 8)	9.85 ± 1.06 (*n* = 15)	0.478
HGB (g/L)	142.25 ± 11.85 (*n* = 8)	145.33 ± 13.73 (*n* = 15)	0.100
HCT (%)	48.20 ± 3.96 (*n* = 8)	49.99 ± 5.60 (*n* = 15)	0.628
MCV (fL)	50.82 ± 0.74 (*n* = 8)	50.73 ± 1.29 (*n* = 15)	0.664
MCH (pg)	15.04 ± 0.30 (*n* = 8)	14.77 ± 10.31 (*n* = 15)	0.061
MCHC (g/L)	295.00 ± 4.41 (*n* = 8)	291.33 ± 7.65 (*n* = 15)	0.195
%NEUT (%)	10.88 ± 5.10 (*n* = 8)	13.13 ± 3.49 (*n* = 15)	0.222
%LYM (%)	77.03 ± 6.94 (*n* = 8)	74.65 ± 4.41 (*n* = 15)	0.326
%MONO (%)	3.58 ± 1.86 (*n* = 8)	4.07 ± 2.07 (*n* = 15)	0.580
%EOS (%)	4.01 ± 4.32 (*n* = 8)	3.73 ± 1.55 (*n* = 15)	0.349
%LUC (%)	4.11 ± 1.04 (*n* = 8)	4.07 ± 3.09 (*n* = 15)	0.245
%BASO (%)	0.40 ± 0.14 (*n* = 8)	0.35 ± 0.14 (*n* = 15)	0.386
PLT (x10E09 cells/L)	523.50 ± 239.64 (*n* = 8)	633.40 ± 273.18 (*n* = 15)	0.350

Data are shown as mean ± SD. The data for RBC, HGB, HCT, MCHC, %EOS, and %LUC were non-normally distributed and equal in variance, and statistical analyses for those data were performed using the nonparametric Mann–Whitney test. The rest of the data were normally distributed and equal in variance, and statistical analyses were performed using Student's *t*-test. The difference between Chr4^Δ70*kb*/Δ70*kb*^ and wild-type (WT) BM transplant groups was considered statistically significant when ^*^P ≤ 0.05. *P* values reaching the statistical difference are presented in bold.

### 3.3. Hematopoietic Chr4^Δ70*kb*/Δ70*kb*^ knockout increased plasma total cholesterol and triglyceride levels in *Ldlr^−/−^ApoB^100/100^* mice

After 12 weeks of HFD, Chr4^Δ70*kb*/Δ70*kb*^ BM recipient mice showed significantly increased plasma total cholesterol (26.91 ± 3.77 mmol/L, *P* = 0.012) and triglyceride (3.31 ± 1.03 mmol/L, *P* = 0.001) levels compared with WT BM recipients in an *Ldlr*^−/−^*ApoB*^100/100^ background (cholesterol 22.35 ± 3.03 mmol/L, triglycerides 1.60 ± 0.57; Table 2). There were no differences in plasma LDL or HDL levels between the groups. In addition, the fasting blood glucose level was increased in Chr4^Δ70*kb*/Δ70*kb*^ BM recipient mice (8.25 ± 0.93 mmol/L) compared with WT BM recipients in the *Ldlr*^−/−^*ApoB*^100/100^ background (7.44 ± 1.09 mmol/L), but the difference was not statistically significant (*P* = 0.054). As the BM-specific deletion of the murine CAD risk locus increased the plasma total cholesterol and triglyceride levels, we measured the expression of master regulators of liver fatty acid and cholesterol synthesis. Interestingly, the hematopoietic Chr4^Δ70*kb*/Δ70*kb*^ significantly increased the hepatic expression of *Fasn* in *Ldlr*^−/−^*ApoB*^100/100^ mouse livers (*P* = 0.048; Supplementary Figure 4A) but did not affect the expression of *Srebf1* (Supplementary Figure 4B) and *Srebf2* (Supplementary Figure 4C) compared with WT BM recipients after 12 weeks of HFD. Nevertheless, the hepatic steatosis and the accumulation of inflammatory cells in the liver were equal between the Chr4^Δ70*kb*/Δ70*kb*^ BM and WT BM transplanted *Ldlr*^−/−^*ApoB*^100/100^ mice after 12 weeks of HFD (Supplementary Figure 4D).

### 3.4. Chr4^Δ70*kb*/Δ70*kb*^ knockout promotes the pro-inflammatory phenotype in bone marrow-derived macrophages

**Figure 4:**
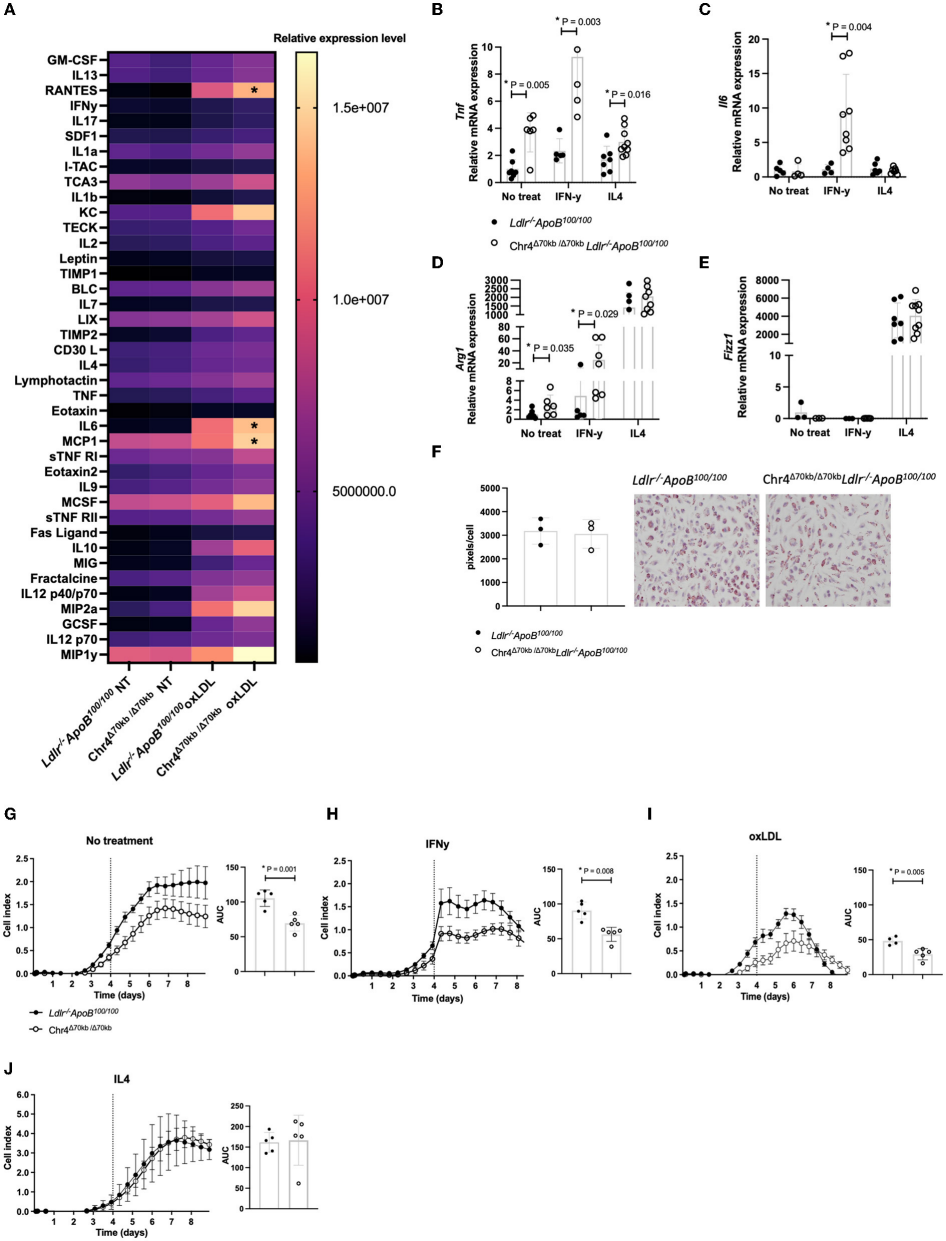
Deficiency in the murine ortholog of 9p21.3 risk locus leads to more pro-inflammatory phenotype and reduced proliferation in *Ldlr*^−/−^*ApoB*^100/100^ BMDMs. **(A)** Cytokine array by oxLDL treatment in Chr4^Δ70*kb*/Δ70*kb*^*Ldlr*^−/−^*ApoB*^100/100^ (*n* = 4) and *Ldlr*^−/−^*ApoB*^100/100^ (*n* = 4) BMDMs. The relative protein level expression of IL6 (*P* = 0.040), MCP1 (*P* = 0.033), and RANTES (*P* = 0.041) were significantly increased in response to oxLDL in Chr4^Δ70*kb*/Δ70*kb*^*Ldlr*^−/−^*ApoB*^100/100^ BMDMs compared with *Ldlr*^−/−^*ApoB*^100/100^ BMDMs. **(B)** Macrophage M1/M2 polarization assay was performed and the mRNA expression of M1 markers Tnf and **(C)** Il6 was measured in *Ldlr*^−/−^*ApoB*^100/100^ (*n* = 4–7) and Chr4^Δ70*kb*/Δ70*kb*^ *Ldlr*^−/−^*ApoB*^100/100^ (*n* = 4–9) BMDMs after 16 h exposure to IFN-γ and IL4. The mRNA expression of macrophage M2 markers **(D)** *Arg1* and **(E)** *Fizz1* in *Ldlr*^−/−^*ApoB*^100/100^ (*n* = 3–7) and Chr4^Δ70*kb*/Δ70*kb*^ *Ldlr*^−/−^*ApoB*^100/100^ (*n* = 3–9) BMDMs after 16 h exposure to IFN-γ and IL4. **(F)** Foam cell assay presenting the average area of Oil Red O stained lipid droplets in Chr4^Δ70*kb*/Δ70*kb*^*Ldlr*^−/−^*ApoB*^100/100^ and *Ldlr*^−/−^*ApoB*^100/100^ BMDMs after oxLDL treatment. **(G)** Proliferation rate of Chr4^Δ70*kb*/Δ70*kb*^*Ldlr*^−/−^*ApoB*^100/100^(*n* = 4–5) *and Ldlr*^−/−^*ApoB*^100/100^ (*n* = 5) BMDMs at the basal level, **(H)** in response to IFN-γ treatment, **(I)** in response to oxLDL treatment, and **(J)** in response to IL4 treatment. Cell proliferation is recorded as a cell index, representing the impedance of the plated cells measured by the xCELLigence^®^ Real-Time Cell Analysis instrument. The vertical line on the x-axis represents the time point of the cell culture media change and administration of the treatment. Graphs show mean ± SD. Measured mRNA levels were normalized to endogenous control *Gapdh* and analysis of relative gene expression levels was made by using the 2–ΔΔCt method. Differences in proliferation were investigated by measuring the area under the curve (AUC) of the cell index from each replicate and comparing the group means of these values. The data in graphs **(B–E, H, J)** were non-normally distributed and had differences in variance, and analysis for statistical significance was performed using the nonparametric Mann–Whitney test. The data in graphs **(G, I)** were normally distributed and had equal variance, and analysis for statistical significance was performed using the Student's *t*-test. *P* ≤ 0.05 was considered a threshold for statistical significance.

As the CAD risk locus and its transcripts may potentially promote atherogenesis *via* inflammatory cell-specific regulation in *Ldlr*^−/−^*ApoB*^100/100^ mice and macrophages are the most prominent inflammatory cell type in mouse atherosclerotic plaques, we extracted BM-derived macrophages (BMDMs) from Chr4^Δ70*kb*/Δ70*kb*^*Ldlr*^−/−^*ApoB*^100/100^ and *Ldlr*^−/−^*ApoB*^100/100^ mice and aimed to clarify the role of the CAD risk locus in macrophage polarization, foam cell formation, and proliferation. Pro-inflammatory, IFN-γ-induced, M1 macrophages are classically considered proatherogenic, whereas IL4-induced M2 macrophages are anti-inflammatory and provide atheroprotective effects. Interestingly, the secretion of several pro-inflammatory cytokines and chemokines, including IL6 (*P* = 0.040), MCP1 (*P* = 0.033), and RANTES, was significantly increased at the protein level in response to oxLDL in Chr4^Δ70*kb*/Δ70*kb*^*Ldlr*^−/−^*ApoB*^100/100^ BMDMs compared with *Ldlr*^−/−^*ApoB*^100/100^ BMDMs (Figure 4A). CXCL1/KC (*P* = 0.053), CXCL2/MIP2a (*P* = 0.08), and CCL9/MIP1y (*P* = 0.051) also showed increased protein-level expression, but without statistical significance. The enhanced pro-inflammatory phenotype was also supported by the macrophage polarization assay as the mRNA expression of M1 marker *Tnf* was already higher at the basal level (*P* = 0.005) and also in response to both IFN-γ (*P* = 0.003) and IL4 (*P* = 0.016) in Chr4^Δ70*kb*/Δ70*kb*^*Ldlr*^−/−^*ApoB*^100/100^BMDMs compared with *Ldlr*^−/−^*ApoB*^100/100^BMDMs (Figure 4B). Moreover, the expression of another M1 marker, *Il6*, was increased at the mRNA level in response to IFN-γ (*P* = 0.004) and was not altered on the basal level or after IL4 treatment in Chr4^Δ70*kb*/Δ70*kb*^*Ldlr*^−/−^*ApoB*^100/100^ BMDMs compared with *Ldlr*^−/−^*ApoB*^100/100^ BMDMs (Figure 4C). The expression of macrophage M2 phenotype markers, *ArgI* and *Fizz1*, was also measured on the basal level and in response to IL4 and IFN-γ in BMDMs. Surprisingly, *ArgI* expression was significantly higher at the basal level (*P* = 0.035) and in response to IFN-γ (*P* = 0.029) in Chr4^Δ70*kb*/Δ70*kb*^*Ldlr*^−/−^*ApoB*^100/100^ BMDMs compared with *Ldlr*^−/−^*ApoB*^100/100^ BMDMs, but expression levels were equal between genotypes in response to IL4 (Figure 4D). *Fizz1* expression in BMDMs was equal between genotypes at the basal level and in response to IL4 and IFN-γ (Figure 4E). However, the enhanced pro-inflammatory activity in Chr4^Δ70*kb*/Δ70*kb*^*Ldlr*^−/−^*ApoB*^100/100^ BMDMs did not have an effect on macrophage cholesterol accumulation and foam cell formation as the BMDMs were treated with oxLDL and the cellular neutral lipid content was measured by Oil Red O, but no differences were observed between the genotypes (Figure 4F). Finally, Chr4^Δ70*kb*/Δ70*kb*^*Ldlr*^−/−^*ApoB*^100/100^ BMDMs showed significantly lower proliferation both at the basal level (*P* = 0.001; Figure 4G) and after IFN-γ (*P* = 0.008; Figure 4H) and oxLDL (*P* = 0.005; Figure 4I) but not after IL4 (Figure 4J) treatment compared with *Ldlr*^−/−^*ApoB*^100/100^ BMDMs.

### 3.5. *ANRIL* is expressed in immune cells in human and mouse atherosclerotic plaques

**Figure 5:**
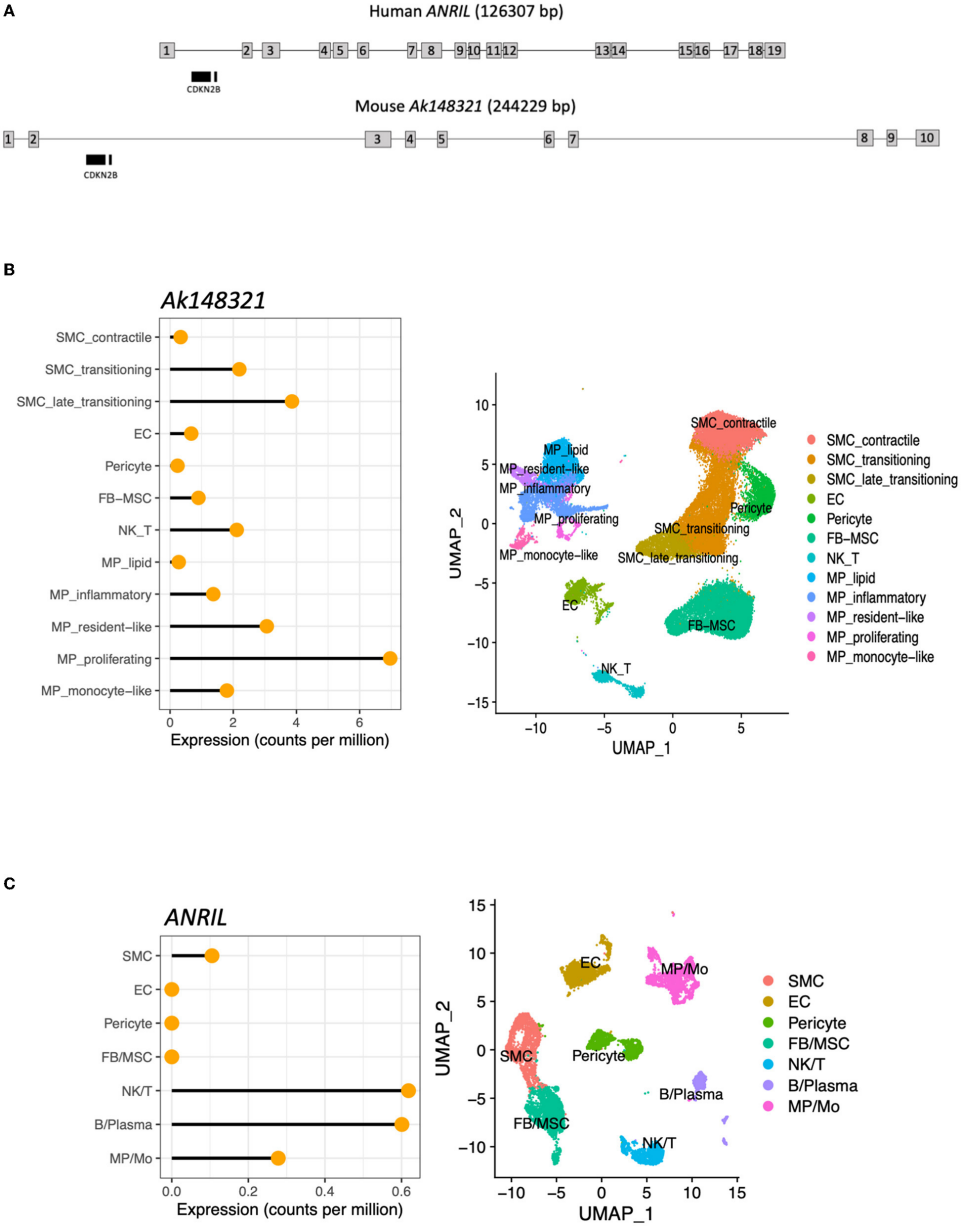
Human *ANRIL* and its murine equivalent, *Ak148321*, are expressed mainly in the immune cells of atherosclerotic plaques. **(A)** Representative schema of human *ANRIL* and mouse *Ak148321* loci, based on human transcript *NR_003529.3* and mouse *Ak148321*.1 as visualized on the UCSC genome browser. No exact match between the human and mouse transcripts has been established. Not to scale. **(B)** Cell type-specific expression of *Ak148321* (RefSeq lncRNA gene *Gm12610* transcript variant NR_132431.1) in mouse atherosclerotic aortas (15). **(C)** Cell type-specific expression of *ANRIL* (*CDKN2B-AS1*) in human coronary atherosclerotic plaques (14). For both **(A, B)**, scRNA-seq cell clusters were manually annotated into cell types or lineages (as described in Section 2), and the average gene expression within each cell population is presented as counts per million.

Because both global and hematopoietic deletions in the murine CAD risk orthologous locus led to increased atherosclerosis, global Chr4^Δ70*kb*/Δ70*kb*^*Ldlr*^−/−^*ApoB*^100/100^ mice showed increased blood leukocyte count, and Chr4^Δ70*kb*/Δ70*kb*^ BMDMs showed more pro-inflammatory phenotype *in vitro*, we decided to investigate if human *ANRIL* and its murine equivalent (Figure 5A) are expressed in inflammatory cell populations in atherosclerotic plaques, and how their expression levels are in comparison with other vascular cell populations. We analyzed public single-cell RNA sequencing data from Pan et al. (15) and found that murine *ANRIL* equivalent *Ak148321* is expressed most in macrophages and SMCs of mouse atherosclerotic aortas (Figure 5B). We were also able to focus on the major macrophage and SMC subtypes, and the results suggest that a proliferating macrophage subpopulation has the highest expression of *Ak148321*. Out of the plaque SMCs, the transitioning SMCs had the highest expression of *Ak148321*. For human atherosclerosis, we analyzed the scRNA sequencing data of human coronary plaques from Wirka et al. (14) and found that *ANRIL* expression was highest in T-cells and B-cells, followed by macrophages (Figure 5C).

### 3.6. HFD modulates the expression of a circular isoform of *ANRIL* equivalent *Ak148321*

**Figure 6:**
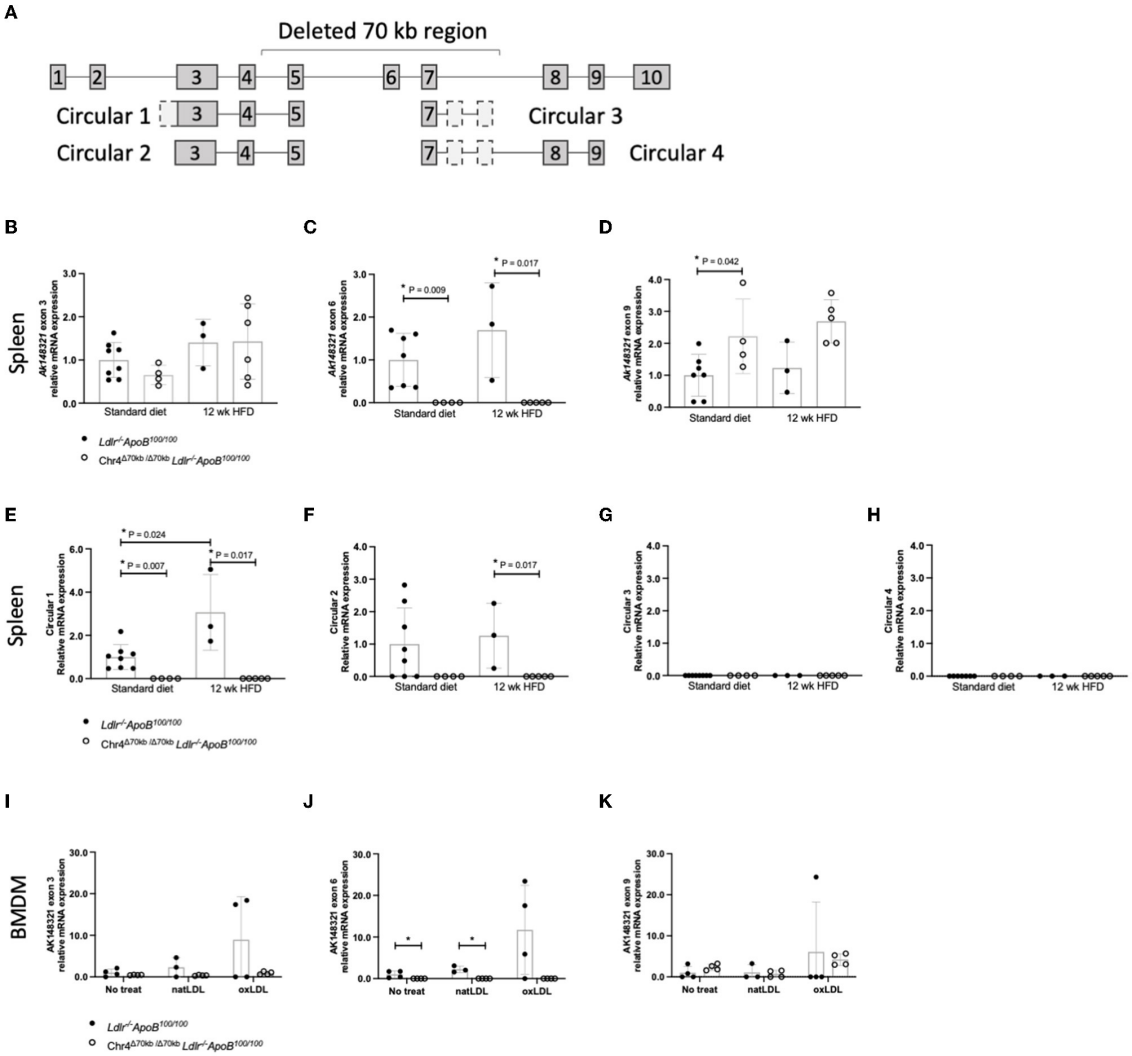
Two of the expected circular *Ak148321* isoforms were confirmed in the spleen of *Ldlr*^−/−^*ApoB*^100/100^ mice, one of which was modulated by HFD. **(A)** Localization of the deletion and circular isoforms on the *Ak148321* (mm9: chr4:88941084-89185312). Gray boxes represent canonical exons of the linear isoform, whereas dashed lines indicate exonic sequence only present in the circular isoform. **(B–D)** Relative mRNA expression of *Ak148321* exons 3, 6, and 9 in *Ldlr*^−/−^*ApoB*^100/100^ (*n* = 7 – 8) and Chr4^Δ70*kb*/Δ70*kb*^ *Ldlr*^−/−^*ApoB*^100/100^ mice (*n* = 4) spleens after the standard laboratory diet and in *Ldlr*^−/−^*ApoB*^100/100^ (*n* = 3) and Chr4^Δ70*kb*/Δ70*kb*^ *Ldlr*^−/−^*ApoB*^100/100^ mice (*n* = 5 – 6) after 12 weeks of HFD. **(E–H)** Relative mRNA expression of *Ak148321* circular isoforms in *Ldlr*^−/−^*ApoB*^100/100^ and Chr4^Δ70*kb*/Δ70*kb*^ *Ldlr*^−/−^*ApoB*^100/100^ mice spleens after the standard laboratory diet (*n* = 8 + 4) and 12 weeks of HFD (*n* = 3 + 4). **(I–K)** Relative mRNA expression of *Ak148321* exons 3, 6, and 9 in *Ldlr*^−/−^*ApoB*^100/100^ BMDMs and Chr4^Δ70*kb*/Δ70*kb*^ *Ldlr*^−/−^*ApoB*^100/100^ BMDMs after 16 h exposure to a 50 μg/ml dose of natLDL (*n* = 3 + 4) or oxLDL (*n* = 4 + 4) compared with nontreated samples (*n* = 4 + 4). Measured mRNA levels were normalized to endogenous control *Gapdh*, and analysis of relative gene expression levels was made using the 2–ΔΔCt method. Graphs show mean ± SD. Statistical analyses were performed using the nonparametric Mann–Whitney test, and the difference between the groups was considered statistically significant when ^*^P ≤ 0.05.

To further examine the expression pattern of the CAD risk locus murine equivalent in an inflammatory cell context, we measured the expression of three *Ak148321* exons and four expected circular isoforms in the spleens of Chr4^Δ70*kb*/Δ70*kb*^*Ldlr*^−/−^*ApoB*^100/100^ and *Ldlr*^−/−^*ApoB*^100/100^ mice on the standard laboratory diet and in response to prolonged HFD (Figure 6A). HFD did not significantly affect the expression of *Ak148321* exons 3, 6, or 9 in the spleens of Chr4^Δ70*kb*/Δ70*kb*^*Ldlr*^−/−^*ApoB*^100/100^ or *Ldlr*^−/−^*ApoB*^100/100^ mice (Figures 6B–D), However, exon 9 expression was significantly higher in the spleens of Chr4^Δ70*kb*/Δ70*kb*^*Ldlr*^−/−^*ApoB*^100/100^ mice compared with *Ldlr*^−/−^*ApoB*^100/100^ mice on standard laboratory diet (*P* = 0.042). Next, we measured the expression of four expected *Ak148321* circular isoforms in response to HFD. Interestingly, the expression of Circular 1 was increased in *Ldlr*^−/−^*ApoB*^100/100^ mouse spleen by HFD (*P* = 0.024; Figure 6E), but HFD had no effect on the expression of Circular 2 (Figure 6F). Circular 3 and Circular 4 remained undetected in *Ldlr*^−/−^*ApoB*^100/100^ mouse spleen, and none of the circular *Ak148321* isoforms were detected in Chr4^Δ70*kb*/Δ70*kb*^*Ldlr*^−/−^*ApoB*^100/100^ mouse spleen (Figures 6G, H). Finally, to mimic the atherosclerotic plaque environment, the expression of three *Ak148321* exons was measured in Chr4^Δ70*kb*/Δ70*kb*^*Ldlr*^−/−^*ApoB*^100/100^ and *Ldlr*^−/−^*ApoB*^100/100^ BMDMs in response to oxLDL. *Ldlr*^−/−^*ApoB*^100/100^ BMDMs showed a tendency to a higher expression of *Ak148321* exons 3 (Figure 6I) and 6 (Figure 6J) in response to oxLDL, but the difference was not statistically significant. As expected by the knockout, *Ak148321* exon 6 was not detected in Chr4^Δ70*kb*/Δ70*kb*^ BMDMs (Figure 6J). There were no differences in the expression pattern of *Ak148321* exon 9 in BMDMs in response to oxLDL or by the Chr4^Δ70*kb*/Δ70*kb*^ (Figure 6K).

### 3.7. The expression of the neighboring genes Cdkn2A and Cdkn2B at the risk locus was not affected by Chr4^Δ70*kb*/Δ70*kb*^ in *Ldlr^−/−^ApoB^100/100^* mice

Two cell cycle regulator genes, *CDKN2A* and C*DKN2B*, neighboring the CAD risk interval have been suggested to play a role in the CAD risk associated with the 9p21.3 risk locus. When analyzing single-cell RNA sequencing data from mouse (15) and human (14), we found that, in mouse aortic atherosclerotic plaques, *Cdkn2a* and *Cdkn2b* were expressed mostly in SMCs (Supplementary Figures 5A, B), while in human coronary plaques, they were mostly expressed in T cells (Supplementary Figures 5C, D). To evaluate the impact of *Cdkn2a* and C*dkn2b* in atherosclerosis and the regulation of macrophage phenotype in the Chr4^Δ70*kb*/Δ70*kb*^*Ldlr*^−/−^*ApoB*^100/100^ mouse model, we measured the expression of *Cdkn2a* and *Cdkn2b* in Chr4^Δ70*kb*/Δ70*kb*^ and *Ldlr*^−/−^*ApoB*^100/100^ BMDMs and the spleens of Chr4^Δ70*kb*/Δ70*kb*^*Ldlr*^−/−^*ApoB*^100/100^ and *Ldlr*^−/−^*ApoB*^100/100^ mice after oxLDL treatment or HFD, respectively. The expression of C*dkn*2a was increased in nontreated Chr4^Δ70*kb*/Δ70*kb*^*Ldlr*^−/−^*ApoB*^100/100^ BMDMs (*P* = 0.029), but not in natLDL or oxLDL-treated Chr4^Δ70*kb*/Δ70*kb*^ BMDMs compared with *Ldlr*^−/−^*ApoB*^100/100^ BMDMs (Supplementary Figure 5E). *Cdkn2b* expression did not significantly differ between Chr4^Δ70*kb*/Δ70*kb*^*Ldlr*^−/−^*ApoB*^100/100^ and *Ldlr*^−/−^*ApoB*^100/100^ BMDMs at the basal level or in response to native or oxLDL treatment (Supplementary Figure 5F). Finally, the splenic expression of *Cdkn2a* was upregulated by the HFD in *Ldlr*^−/−^*ApoB*^100/100^ mice (*P* = 0.024), while in Chr4^Δ70*kb*/Δ70*kb*^*Ldlr*^−/−^*ApoB*^100/100^ spleens, the expression was not modulated by diet (Supplementary Figure 5G). There were no differences in the expression of *Cdkn2a* or *Cdkn2b* in the spleen of Chr4^Δ70*kb*/Δ70*kb*^*Ldlr*^−/−^*ApoB*^100/100^ mice compared with *Ldlr*^−/−^*ApoB*^100/100^ mice on the standard laboratory diet or after 6 or 12 weeks of HFD (Supplementary Figures 5G, H).

## 4. Discussion

The Chr9p21.3 risk locus and its lncRNA transcript, *ANRIL*, have been identified as the most significant genetic risk region for atherosclerotic cardiovascular disease, independently of conventional risk factors like hyperlipidemia (5). Several SNPs at the region are linked to the expression of *ANRIL* and its linear and circular splicing isoforms. The risk locus has also been reported to be associated with aneurysms (18), type 2 diabetes (19), and a variety of cancers, including melanoma (20), glioma (21), and breast cancer (22). In this study, we discovered that the deletion of the murine orthologous locus to the Chr9p21.3 CAD risk interval promotes advanced atherosclerosis, possibly in a hematopoietic cell-dependent manner by regulation of circulating leukocyte number and the pro-inflammatory activity of macrophages in hypercholesterolemia-stressed conditions. Surprisingly, hematopoietic Chr4^Δ70*kb*/Δ70*kb*^ also elevated plasma total cholesterol and triglyceride levels in *Ldlr*^−/−^*ApoB*^100/100^ mice, which potentially also accelerated the development of atherosclerotic plaques.

### 4.1. The deletion of *Ak148321*, the murine ortholog of human *ANRIL*, promotes atherosclerosis

Several mechanisms for how *ANRIL* and its splicing isoforms affect atherogenesis have been proposed. In human studies, circular *ANRIL* isoforms have been linked to possible protection against CAD, while linear isoforms are considered pro-atherogenic (6). In the present study, we demonstrated for the first time two circular RNA isoforms being expressed from the CAD risk orthologous locus in mice. Considering the multiple isoforms of *ANRIL* and its murine equivalent, *Ak148321*, both disruption of the full-length linear transcript and deficiency of the potentially protective circular transcripts are considered to mediate the proatherogenic effect of Chr4^Δ70*kb*/Δ70*kb*^ in mice. Moreover, the increased expression of one of the circular isoforms in the spleen by HFD further suggests the activation of the circular isoforms under metabolic stress. Holdt et al. reported that *circANRIL* plays a role in vascular cell proliferation and apoptosis, showing an association between higher expression of *circANRIL*, stronger induction of apoptosis, and reduced proliferation in human SMCs, as well as demonstrating how *circANRIL* acts as a binding component of proteins regulating ribosomal RNA maturation (6).

A previous study using *ApoE*^−/−^ mice (9) has concluded that the deletion of the orthologous CAD risk locus in mice increases the size of advanced atherosclerotic plaques, with no other effects on plaque vulnerability than increased plaque calcification. That study proposed that the deletion of the risk locus accelerates vascular SMC proliferation, the expression of the calcification-regulating genes, and the sensitivity to ossification. In line with these reports, we indicated that both total and hematopoietic deletion of the risk locus ortholog in mouse models increases the development of advanced but not early atherosclerotic plaques. Interestingly, we did not detect any major changes in plaque morphology or stability in early or advanced atherosclerosis. However, the increased proportion of SMCs found in atherosclerotic plaques of hematopoietic Chr4^Δ70*kb*/Δ70*kb*^ mice is in line with the previous study on *ApoE*^−/−^ mice. Pro-inflammatory activation is known to promote SMC migration and proliferation in the vascular wall (23). Nevertheless, only a small number of SMCs were present in both early and advanced murine atherosclerotic plaques in this study and thus possibly have only a minor contribution to plaque development.

### 4.2. *ANRIL* and *Ak148321* regulate the inflammatory cell function in human and mouse atherosclerosis

Despite their structural differences, the single-cell sequencing analysis from atherosclerotic plaques demonstrated the expression of both murine *Ak148321* and human *ANRIL* being highest in macrophages and T- and B-cells. In this study, the number of plaque lymphocytes was remarkably low in both systemic and hematopoietic Chr4^Δ70*kb*/Δ70*kb*^ mouse plaques, leaving macrophages as the dominant inflammatory cell type. The Chr4^Δ70*kb*/Δ70*kb*^ BMDMs showed a more pro-inflammatory phenotype *in vitro*, with elevated expression of *Il6* and other pro-inflammatory cytokines and chemokines after exposure to oxLDL and IFN-γ. The vascular pro-inflammatory activity regulated by *ANRIL* proposed in the present study is also supported by others, as macrophages from MI patients carrying the homozygous risk haplotype have been reported to express increased levels of pro-inflammatory chemokines MCP-1 and −2 compared with patients with the nonrisk haplotype (24). *ANRIL* has also been reported to regulate the inflammatory response in human endothelial cells by direct binding with Yin Yang 1 (*YY1*) and thereby regulating the NF-κB pathway and other inflammatory genes, such as *IL6* and *IL8*, downstream of the *TNF* pathway (7). In our study, the proliferation rate of macrophages was reduced by Chr4^Δ70*kb*/Δ70*kb*^ at the basal level and in response to oxLDL and IFN-γ, which may indicate increased apoptosis in macrophages and, potentially, increased secondary inflammation, as also proposed by Holdt et al. (6). To conclude, the biology of the risk locus transcripts in the regulation of macrophage function and atherogenesis seem s to be at least partially similar between human and mouse models, and it may explain the higher susceptibility to atherosclerosis in hypercholesterolemic Chr4^Δ70*kb*/Δ70*kb*^ mice and human carriers of the risk SNPs.

### 4.3. Hematopoietic Chr4^Δ70*kb*/Δ70*kb*^ leads to a metabolic phenotype in *Ldlr^−/−^ApoB^100/100^* mice

Interestingly, the hematopoietic Chr4^Δ70*kb*/Δ70*kb*^ in hypercholesterolemic mice elevated plasma total cholesterol and triglyceride levels after 12 weeks of HFD, and that potentially at least partially accelerated the development of the atherosclerotic lesion. However, as this elevation was not observed in the systemic Chr4^Δ70*kb*/Δ70*kb*^*Ldlr*^−/−^*ApoB*^100/100^ mice that have a deletion in all tissues, including the master lipid metabolism regulating the organs such as the liver, it is likely causing the slightly different metabolic phenotype compared with hematopoietic Chr4^Δ70*kb*/Δ70*kb*^ mice. Moreover, lipid metabolism is essential for the inflammatory response, and both enhanced pro-inflammatory stimuli and low-grade inflammation are known to promote liver *de novo* lipogenesis. Therefore, the elevated plasma lipid levels in hematopoietic Chr4^Δ70*kb*/Δ70*kb*^ mice are most likely a secondary effect of increased pro-inflammatory activity (25). In addition, hematopoietic Chr4^Δ70*kb*/Δ70*kb*^ surprisingly increased the expression of the master regulator of fatty acid synthesis, *Fasn*, which possibly reflected elevated plasma triglyceride and cholesterol levels but did not significantly increase the expression of *Srebf* s in the liver of *Ldlr*^−/−^*ApoB*^100/100^ mice. These mice also showed other metabolic changes, like increased percentual body weight gain, but the body weight did not differ between the groups in the end after 12 weeks of HFD. Plasma fasting glucose levels were also elevated in hematopoietic Chr4^Δ70*kb*/Δ70*kb*^ mice, but the difference did not reach statistical significance. It is notable that the systemic Chr4^Δ70*kb*/Δ70*kb*^ mice also had higher plasma fasting glucose levels compared with wild-type mice in *Ldlr*^−/−^*ApoB*^100/100^ background on a standard laboratory diet. Nonetheless, they did not show any difference in the development of atherosclerosis on the standard diet, and this possible metabolic change was not observed between genotypes after 6 or 12 weeks of HFD, possibly due to massive lipid overload and obesity in both controls and Chr4^Δ70*kb*/Δ70*kb*^ mice. These features together indicate a metabolic phenotype by hematopoietic Chr4^Δ70*kb*/Δ70*kb*^ in hypercholesterolemic mice, but further studies are required to clarify the mechanism and whether it is associated with the activation of the immune response.

### 4.4. Neighboring genes, which act as major cell cycle regulators, *Cdkn2a* and *Cdkn2B*, are not affected by Chr4^Δ70*kb*/Δ70*kb*^ in mice

Since there are two major tumor suppressor genes, *CDKN2A* and *CDKN2B*, neighboring the CAD risk interval, the atherogenic effects of the risk locus mediated by *ANRIL* regulating the expression of these genes were observed. The monocyte-specific *Cdkn2a* deficiency has been previously reported to lead to accelerated atherosclerosis, induced pro-inflammatory gene expression, and increased proliferation of macrophages in mice (26). In our study, the pro-inflammatory and pro-atherogenic stimuli were not mediated *via* reduced expression of *Cdkn2a* or *Cdkn2b* as the expressions of *Cdkn2a* and *Cdkn2b* were not changed by Chr4^Δ70*kb*/Δ70*kb*^ in mouse spleen or BMDMs in response to oxLDL. Instead, the basal expression of *Cdkn2a* was increased, and consistently, the proliferation rate decreased in Chr4^Δ70*kb*/Δ70*kb*^ BMDMs. Parallel to our study, Holdt et al. reported that c*ircANRIL* acts independently of *CDKN2A/B, cis-*regulation of the locus, and miRNA sponging, and they also demonstrated that *linANRIL* is unaffected by *circANRIL* expression (6).

### 4.5. Study limitations

The pro-atherogenic and pro-inflammatory effects of Chr4^Δ70*kb*/Δ70*kb*^ observed in this study can be derived either or both from the lack of the potentially protective murine circular *Ak148321* isoforms or from the disruption of the full-length linear isoform. However, the study was not able to specify the role of different murine or human isoforms in atherogenesis and macrophage polarization. In addition, the role of different *ANRIL* and *Ak148321* isoforms in NF-κB-signaling in inflammatory cells and the question of how other cell types in the arterial wall are affected by *ANRIL* and *Ak148321* isoforms in response to pro-atherogenic stimuli require further studies.

### 4.6. Conclusion

To the best of our knowledge, our study demonstrates for the first time the hematopoietic cell-specific effect of the murine ortholog of the human 9p21.3 CAD risk locus and its murine circular isoforms on atherosclerosis in hypercholesterolemic mice. Even though in humans, the pro-atherogenic effects of 9p21.3 are driven by the risk SNPs rather than a full deletion in the locus, the Chr4^Δ70*kb*/Δ70*kb*^*Ldlr*^−/−^*ApoB*^100/100^ mouse model represents similar pro-atherogenic and pro-inflammatory consequences as seen in humans. The pro-atherogenic and pro-inflammatory phenotype of hematopoietic knockout, along with the single-cell sequencing data from both human and mouse models, tracks down the mechanism for the regulation of inflammatory cell functions.

## Data availability statement

Publicly available datasets were analyzed in this study. This data can be found here: https://www.ncbi.nlm.nih.gov/, GSE131780 and GSE155513.

## Ethics statement

The animal study was reviewed and approved by Regional State Administrative Agency (Finland).

## Author contributions

SK, A-KR, TÖ, TS, JH, MUK, NL-K, and SY-H: conception and design or analysis and interpretation of data or both. SK, A-KR, and SY-H: drafting of the manuscript or revising it critically for important intellectual content. All authors: final approval of the manuscript submitted.
