# Supplementary material for: Deletion of the murine ortholog of human 9p21.3 locus promotes atherosclerosis by increasing macrophage proinflammatory activity
Source: Front Cardiovasc Med. 2023 Mar 6;10:1113890. doi: 10.3389/fcvm.2023.1113890 (PMC10025322; doi:10.3389/fcvm.2023.1113890)
Supplement: Supplementary file 1 [file Data_Sheet_1.docx]

**SUPPLEMENTAL MATERIALS**

**MATERIALS AND METHODS**

**1. Mice**

The Chr4^Δ70kb/Δ70kb^ mouse strain in 129S6/SvEvTac background (129S6/SvEvTac-Del(4C4-C5)1Lap/Mmucd, RRID: MMRRC_032091-UCD) was obtained from Mutant Mouse Resource & Research Center (MMRRC). Mice were backcrossed into *Ldlr^-/-^ApoB^100/100^* (B6;129S-Ldlrtm1Her Apobtm2Sgy/J) mouse strain originating from The Jackson Laboratory, as well as into C57Bl/6JOlaHsd (Envigo). *Ldlr^-/-^ApoB^100/100^* strain was chosen as a background strain for due to its similar lipoprotein profile to hyperlipidemic humans, characterized by the high LDL fraction^27^. Chr4^Δ70kb/Δ70kb^Ldlr^-/-^ApoB^100/100^ female mice and their littermate *Ldlr^-/-^ApoB^100/100^* mice were studied on Western type high fat diet (TD.88137, Envigo, 42% of calories from fat and 0.15% cholesterol) for 6 weeks (n=9+10), or 12 weeks (n=12+12). In addition, Chr4^Δ70kb/Δ70kb^*Ldlr^-/-^ApoB^100/100^* mice and their littermate *Ldlr^-/-^ApoB^100/100^* mice were fed a standard laboratory diet (n=6+8) (Teklad Global 16% Protein Rodent Diet: 12% of calories from fat and 0% cholesterol) until the age of 6 months. In the BM transplantation experiment we used male Chr4^Δ70kb/Δ70kb^C57Bl/6JOlaHsd mice (n=3) and their male C57Bl/6JOlaHsd littermates (n=3) as donor animals, and female *Ldlr^-/-^ApoB^100/100^* mice (n=19+11) as recipients. These numbers represent the total number of animals used to obtain samples in this study. The exact sample and replicate numbers used for each individual analysis are stated along with the results, figure legends and tables. Female mice were used in this study due to their increased susceptivity to develop atherosclerosis in comparison to males^28^. For the bone marrow transplantation, male mice were used as donors and female mice as recipients, as this provides a possibility to evaluate the success of transplantation by using Y-chromosome as a marker.

Mice were housed in the Animal Centre of University of Eastern Finland under controlled conditions for temperature and humidity, using a 12 h light/dark cycle and received food and tap water *ad libitum*. When the study period ended, mice were sacrificed with carbon dioxide, perfused with phosphate-buffered saline (PBS), and their tissues were collected. All animal experiments were approved by the National Experimental Animal Board of Finland and carried out following the guidelines of the Finnish Act on Animal Experimentation and Directive 2010/63/EU of the European Parliament. The study was designed and performed according to the American Heart Association guidelines^29^.

**2. Single-cell RNA-Seq (scRNA-Seq) data processing**

For human atherosclerotic plaque scRNA-Seq, data generated in Wirka et al. 2019 ^30^ from coronary arteries was used. The cell barcode–UMI count matrix was obtained from NCBI GEO (accession GSE131778) and processed using the workflow recommended for scRNA-Seq data by the authors of the Seurat package (version 3.1 ^31^), running under R version 3.5 (The R Foundation for Statistical Computing). In total, this provided approximately 11,000 cells and a median of approximately 4,000 UMI counts per cell. Resulting cell clusters were annotated into cell types or lineages using the following marker genes: *PTPRC/CD45* (immune cells), *PECAM1/CD31* and *CDH5* (endothelial cells), *MYH11*, *ACTA2* and *CNN1* (smooth muscle cells), *CD68*, *LYZ2* and *S100A8* (macrophages and monocytes), *CD3D*, *CD8A* and *NKG7* (T and NK cells), *CD79A* (B and plasma cells), *RGS5* and *RGS16* (pericytes), *SFRP2* and *DCN* (fibroblasts and mesenchymal stromal cells), *GPM6B* (neuronal cells), and *CPA3* (mast cells).

For mouse atherosclerosis scRNA-Seq, the raw sequencing reads generated in Pan et al. 2020 ^32^ from mouse aortas were obtained from NCBI GEO (accession GSE155513) and reprocessed using the cellranger count pipeline (version 6.0; 10x Genomics) with a custom reference package and with the option to include intronic counts. Cellranger mkref (version 6.0; 10x Genomics) was used to generate the custom reference package, in which the gene definitions of the mouse mm10 reference package version 2020-A (10x Genomics) were modified to include the NCBI RefSeq transcript for the gene Gm12610 (RefSeq accession NR_132431.1, based on *Ak148321*) and the overlapping Ensembl gene definitions for Gm12610 and Gm12609 were removed. To exclude low-quality cells and potential multiplet events, cell barcodes were required to have 500–4,000 genes detected, 1,000–20,000 UMI counts and <10% mitochondrial UMI-s. In total, this provided approximately 42,000 cells and a median of approximately 6,600 UMI counts per cell. The cell data was processed using the standard Seurat version 3 RNA processing workflow, followed by integration using harmony (version 1.0^33^) with default parameters. After integration, the cells were grouped into clusters using Seurat FindClusters with resolution 1.0, and the resulting clusters were manually assigned to cell types or lineages. The same marker genes as for human scRNA-Seq (listed above) were used, with additional markers to classify abundant cell subtypes more specifically: *Trem2* (lipid-associated macrophages), *Il1b* (inflammatory macrophages), *Cd163* (resident-like macrophages), *Stmn1* (proliferating macrophages), *S100a8* (monocyte-like), *Ly6a* (transitioning SMC), and *Fmod* (late transitioning SMC). For both mouse and human, gene expression level by cell type was calculated using the AverageExpression function of Seurat across all cells assigned to the classification and the results were depth-normalized to obtain the UMI counts per million. Rare cell types, with <500 cells available, were excluded (for human, neuronal and mast cells; for mouse, neuronal and mesothelial cells).

**3. Bone marrow transplantation**

Prior to BM transplantation, *Ldlr^-/-^ApoB^100/100^* female recipient mice (n=30) were irradiated with a clinical linear accelerator (Elekta Infinity, Elekta AB, Stockholm, Sweden). 6 MV photon irradiation was used twice at a dose of 5.5 Gy, resulting in a total dose of 11 Gy ^34^. Chr4^Δ70kb/Δ70kb^ (n=3) and WT male mice (n=3) were used as donors. Donor mice were sacrificed with CO_2_, and their BM was harvested by flushing sterile 0.9% NaCl through the collected femur and tibia bones. 24 h after the irradiation, Chr4^Δ70kb/Δ70kb^ (n=19) or WT (n=11) BM was transplanted into recipients by injecting 1.3 x 10^7^ cells in 200 µl of 0.9% NaCl to each recipient mouse via tail vein ^34.^ Injections were done under isoflurane inhalation anesthesia. Mice had a 4-week recovery period in sterilized cages with autoclaved water and chow diet after the transplantation. After the recovery, mice were put on HFD for 12 weeks, after which they were sacrificed and sampled for histology and gene-expression analyses as described below. Success of the transplantation was assessed from peripheral leucocyte DNA extracted from whole blood samples (see below*; 4. Assessment of chimerism*). After that, two mice were excluded from each group due to the contradicting PCR results, leaving 17 mice to the Chr4^Δ70kb/Δ70kb^ BM transplant group and 9 in WT BM transplant group.

**4. Assessment of chimerism**

Genomic DNA was isolated from peripheral leukocytes by incubating 200 μl whole blood with 200μl in QuickExtract™ DNA Extraction Solution (Lucigen) at 64 ˚C for 10 min and 2 min at 98 ˚C. The genomic CAD risk interval deletion was amplified by PCR using primers annotated by Visel et al 2010.^35^ PCR amplifications were performed in a final volume of 25 μl containing 2 µl of template DNA solution, 1 μl (10 mM) dNTP, 5 μl 5 x buffer containing 1.5 mM of MgCl_2_, 1.7 μl (25 nM) MgCl_2,_ 1 μl of each primer, 0.5 μl Phire DNA polymerase (Thermo Scientific) and 10.8 μl H_2_O. After 2 min denaturation at 98 ˚C, samples were subjected to 30 cycles at 98 ˚C for 20 sec, 58 ˚C for 20 sec and 72 ˚C for 20 sec, followed by 5 min final extension at 72 ˚C. The PCR amplicons were electrophoresised in 1.5% agarose gel at 120 V for 60 min. The resulting 180 bp (wild-type) and 236 bp (deletion) bands were visualized using Gel Doc XR (BioRad).

**5. Histology**

Histological samples were fixed in 4% formaldehyde in PBS (pH 7.4) overnight and then embedded in paraffin. After that, tissues were cut into 4 µm thick sections and stained. Images were analyzed with Fiji software ^36^. Atherosclerosis was analyzed from cross-sections of aortic roots. For the analysis of atherosclerotic plaque area, the aortic root cross-sections (mean of 3 sections per mouse at 40μm intervals) were stained with hematoxylin-eosin ^37,38^. To analyze plaque composition, aortic root sections were immuno-stained with MAC3 primary antibody (rat-anti mouse, BD Pharmingen™) to measure plaque macrophage content, CD3e primary antibody (Rabbit IgG, Cell Signaling Technology) for plaque lymphocytes and with anti-a-smooth muscle actin (Sigma-Aldrich) for SMC content. Plaque collagen and fibrosis were stained by Sirius Red S, respectively ^36^. Necrosis was measured from the Masson trichrome (Sigma-Aldrich) stained cross-sections. Plaque composition parameters were measured in relation to total plaque area, by using color threshold tool (Fiji-software). All variables were measured from two to three different sections per mouse and their average was calculated. All the analyses were done in a blinded manner.

**6. Blood analysis**

Mice were fasted for 4 h prior to sampling. Blood fasting glucose was measured before sacrification from the tail vein, using Ascencia Elite XL glucose meter (Bayer). Blood samples were collected right after sacrification with cardiac puncture and blood was transferred into EDTA tubes. Blood count was analyzed from whole blood, while prior to lipid analyses samples were centrifuged for plasma separation. Blood count, total cholesterol, LDL, HDL, and triglyceride levels were measured by veterinary laboratory services provided by Movet Oy (Kuopio, Finland).

**7. BMDM experiments**

***7.1. Culturing of bone marrow derived macrophages***

Bone marrow cells were isolated from the femurs and tibias of Chr4^Δ70kb/Δ70kb^ *Ldlr^-/-^ApoB^100/100^* and *Ldlr^-/-^ApoB^100/100^* mice ^34.^ The cells were incubated in RPMI-medium with macrophage colony stimulating factor (10% FBS, 1% Penicillin/Streptomycin and 20 ng/ml VWR SHBT200-08 M-CSF in 500 ml RPMI). The resulting BMDMs were plated in 12 well plate (800 000 cells/well) and treated with native LDL (50 µg/ml), oxLDL (50 µg/ml), IFN-γ (20ng/ml) or IL4 (20ng/ml) for 16 h. After that, cells were harvested with TRI Reagent™ for RNA-extraction.

***7.2. LDL modification and foam cell assay***

Human LDL solution (VWR International Ltd) was oxidized by incubating LDL with 20 µM concentration CuSO_4_ for 24 h ^39^. To compare the ability of macrophages to take up modified LDL, BMDMs were plated on chamber slides (BD Falcon^TM^) and grown for 4 h in Optimem (Gibco®) with 10% LPDS. Then BMDMs were incubated for 12 h at + 37 ˚C in the absence or presence of native or oxLDL (50 µg/ml). Cells were fixed with 4% PFA and stained with Oil Red O. The total lipid area per cell was calculated by Image J 1.48V based on the threshold.

***7.3. Cytokine array***

Forty inflammatory factors were measured from BMDM cell culture media of 4 Chr4^Δ70kb/Δ70kb^ *Ldlr^-/-^ApoB^100/100^* and 4 *Ldlr^-/-^ApoB^100/100^* No-Treat or oxLDL treated wells by using Mouse Inflammation Antibody Array (ab133999, Abcam). We used protocol described in array manual, with overnight incubations. Chemiluminescence was detected by using ChemiDoc MP imaging system (Bio-Rad Laboratories, Inc.) and signal intensity was quantified by using Image Lab™ Software version 6.0.0 (Bio-Rad).

***7.4 Polarization assay***

BMDMs M1/M2 polarization was analysed by measuring the mRNA expression of *Tnf*, *Il6*, *Arg1* and *Fizz1* in Chr4^Δ70kb/Δ70kb^*Ldlr^-/-^ApoB^100/100^* and *Ldlr^-/-^ApoB^100/100^* BMDMs in response to IFNy and IL4 exposure. RNA extraction, cDNA synthesis and qPCR gene expression analysis were performed as described in chapter 8.

***7.9 Cell proliferation assay***

Proliferation rate of BMDMs was measured with xCELLigence® Real Time Cell Analysis (RTCA) system (ACEA Biosciences Inc.). 25 000 cells well were plated in each well of a specific electronic microplate (E-plate 16) (ACEA Biosciences Inc.) and cultured in xCELLigence® RTCA DP instrument in +37 ˚C for 4 days. Cells were cultured in RPMI medium with M-CSF (RPMI + 10% FBS + 1% P/S + 20 ng/ml M-CSF). On the day four the cells were dosed with 50 µg/ml oxLDL (n=4+5) or 20ng/ml IFNy (n=5+5) or 20ng/ml IL4 (n=5+5), and their proliferation rate was followed for further 7 days. The xCELLigence® instrument monitors the cell proliferation rate in real time by measuring the impedance of plated cells, which increases when more cells are adhered on the microelectrodes located in the bottom of E-plate. Increase in cell number is recorded as cell index, a parameter representing the relative change in impedance. The method is non-invasive and requires no interventions by the researcher, limiting the chance of human error and handling related cell loss.

**8. Gene expression analysis**

Tissues harvested for gene-expression analyses were immediately placed in liquid nitrogen in tubes and stored in -70 ˚C freezers. Total RNA was extracted from the cells and tissues with TRI Reagent™ Solution (Invitrogen™). To remove any contaminating DNA, RNA samples were DNase treated with DNA-free™ DNA Removal Kit (Invitrogen™). After that, RNA was reverse transcribed into cDNA using RevertAid First Strand cDNA Synthesis Kit (Invitrogen™) and random hexamer primers (Promega). Expression levels of different genes were assessed by quantitative Polymerase Chain reaction (qPCR) ran on StepOnePlus™ Real-Time PCR System (Applied Biosystems) using TaqMan™ Universal PCR Master Mix (Applied Biosystems) and TaqMan based assays including custom designed assays for mouse lncRNA *Ak148321* exons 3, 6 (deleted from the Chr4^Δ70kb/Δ70kb^ mice) and 9, as well as for four circular transcripts ^40, 41^ predicted to be disturbed by the knockout (Supplemental Table 1). All assays were acquired from Integrated DNA Technologies and Thermo Fisher Scientific. The measured mRNA levels were normalized to endogenous control *Gapdh*, and relative gene expression levels were analyzed by using 2^–∆∆Ct^ method.

**9. Statistical analysis**

Statistical analyses were performed by using GraphPad Prism version 9.1.0. (GraphPad Software Inc.). Normality of the data was tested by Kolmogorov-Smirnov (KS) normality test and equality of variances by F test. Data was considered non-normally distributed when KS P < 0.05. Variances were considered significantly different when F P < 0.05.

For normally distributed data having equal variances, Student-T test was used for testing the difference of means between test and control group. For the data having non-normal distribution and/or significantly different variances, non-parametric Mann Whitney test was used. Mann Whitney test was also used for the data with n ≤ 5. For comparisons in histology between and within age matched test and control groups on chow and high fat diet, analysis of variance (ANOVA) with Turkey’s multiple comparisons test was performed. Differences were considered statistically significant when P ≤ 0.05. All charts represent data as mean ± SD.

**References**

27. Véniant MM, Beigneux AP, Bensadoun A, Fong LG, Young SG. Lipoprotein size and susceptibility to atherosclerosis--insights from genetically modified mouse models. *Current drug targets*. 2008;9(3):174–89.

28. Mansukhani NA, Wang Z, Shively VP, Kelly ME, Vercammen JM, Kibbe MR. Sex differences in the LDL receptor knockout mouse model of atherosclerosis. *Artery Research*. 2017;20(C):8.

29. Daugherty A, Tall AR, Daemen MJAP, et al. Recommendation on Design, Execution, and Reporting of Animal Atherosclerosis Studies: A Scientific Statement from the American Heart Association. *Arteriosclerosis, Thrombosis, and Vascular Biology*. 2017;37(9):e131–e157.

30. Wirka RC, Wagh D, Paik DT, et al. Atheroprotective roles of smooth muscle cell phenotypic modulation and the TCF21 disease gene as revealed by single-cell analysis. *Nature Medicine*. 2019;25(8):1280–1289.

31. Stuart T, Butler A, Hoffman P, et al. Comprehensive Integration of Single-Cell Data. *Cell*. 2019;177(7):1888-1902.e21.

32. Pan H, Xue C, Auerbach BJ, et al. Single-Cell Genomics Reveals a Novel Cell State during Smooth Muscle Cell Phenotypic Switching and Potential Therapeutic Targets for Atherosclerosis in Mouse and Human. *Circulation*. 2020:2060–2075.

33. Korsunsky I, Millard N, Fan J, et al. Fast sensitive and accurate integration of single-cell data with Harmony. *Nature Methods*. 2019;16(12):1289–1296.

34. Ruotsalainen AK, Inkala M, Partanen ME, et al. The absence of macrophage Nrf2 promotes early atherogenesis. *Cardiovascular Research*. 2013;98(1):107–115.

35. Visel A, Zhu Y, May D, et al. Targeted deletion of the 9p21 non-coding coronary artery disease risk interval in mice. *Nature*. 2010;464(7287):409–412.

36. Schindelin J, Arganda-Carreras I, Frise E, et al. Fiji: An open-source platform for biological-image analysis. *Nature Methods*. 2012;9(7):676–682.

37. Theelen TL, Lappalainen JP, Sluimer JC, et al. Angiopoietin-2 blocking antibodies reduce early atherosclerotic plaque development in mice. *Atherosclerosis*. 2015;241(2):297–304.

38. Ruotsalainen AK, Lappalainen JP, Heiskanen E, et al. Nuclear factor E2-related factor 2 deficiency impairs atherosclerotic lesion development but promotes features of plaque instability in hypercholesterolaemic mice. *Cardiovascular Research*. 2019;115(1):243–254.

39. Ylä-Herttuala S, Palinski W, Rosenfeld ME, et al. Evidence for the presence of oxidatively modified low density lipoprotein in atherosclerotic lesions of rabbit and man. *Journal of Clinical Investigation*. 1989;84(4).

40. Rybak-Wolf A, Stottmeister C, Glažar P, et al. Circular RNAs in the Mammalian Brain Are Highly Abundant, Conserved, and Dynamically Expressed. *Molecular Cell*. 2015;58(5):870–885.

41. Xia S, Feng J, Lei L, et al. Comprehensive characterization of tissue-specific circular RNAs in the human and mouse genomes. *Briefings in Bioinformatics*. 2016;18(6):984–992.

**SUPPLEMENTAL FIGURES**

**
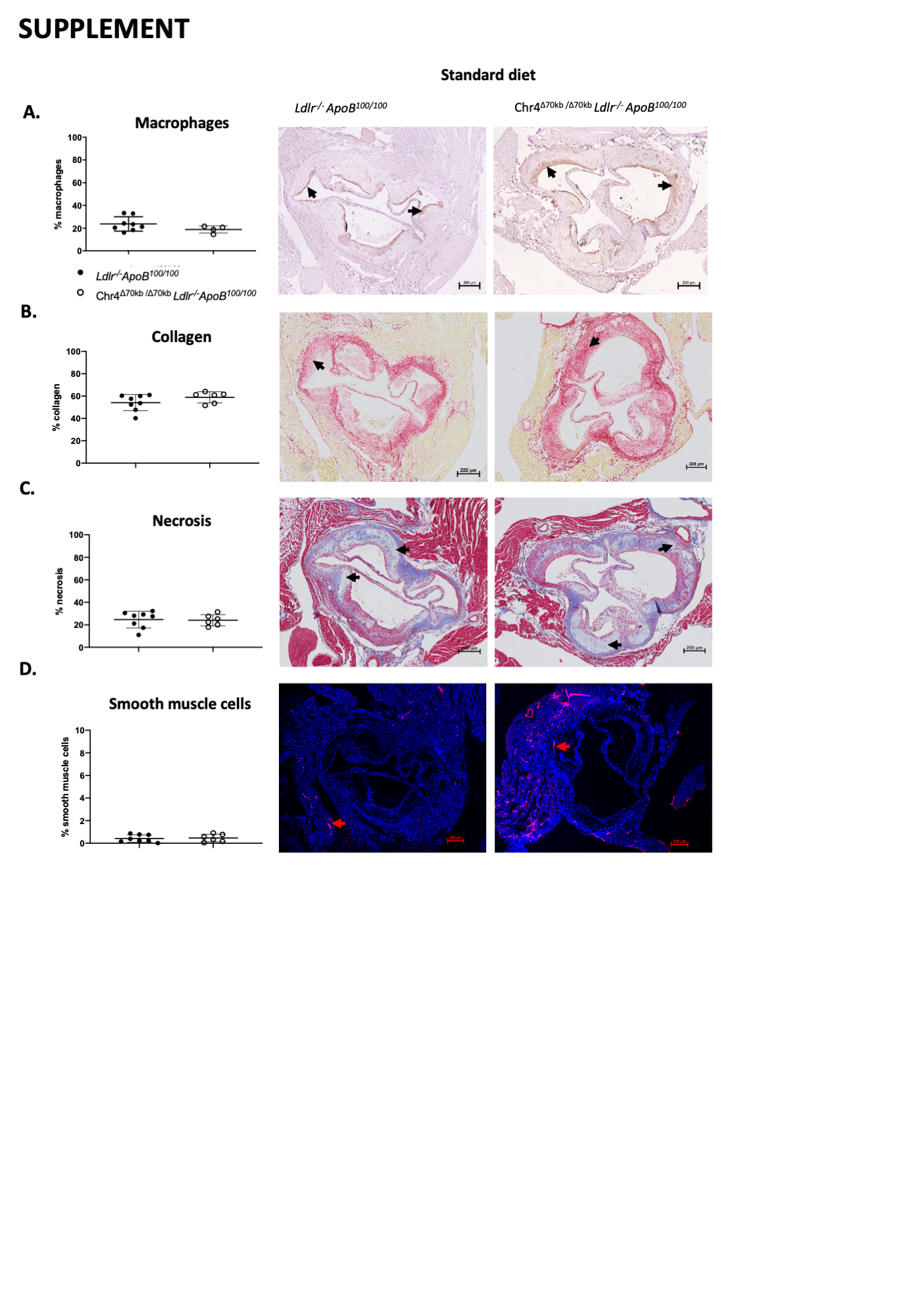
**

**Supplemental Figure 1. There was no statistically significant difference in aortic root plaque morphology between Chr4^Δ70kb /Δ70kb^*Ldlr^-/-^ApoB^100/100^* and *Ldlr^-/-^ApoB^100/100^* mice on the standard laboratory diet.**

**A)** MAC3 positive % area of Chr4^Δ70kb/Δ70kb^*Ldlr^-/-^ApoB^100/100^* (n=4) and *Ldlr^-/-^ApoB^100/100^* (n=8) mice representing macrophages, **B)** Sirius red staining % representing collagen (n=6+8), **C)** Necrotic area % analysed from Masson’s Trichrome staining (n=6+8) and **D)** Fluorescent aSMA positive % area representing smooth muscle cells with DAPI counterstain for nucleus (n=6+8) in aortic root cross sections after 12 weeks standard laboratory diet.

Graphs show mean ± SD. Statistical analyses were performed using Student t test and difference between the groups was considered statistically significant when * P ≤ 0.05.


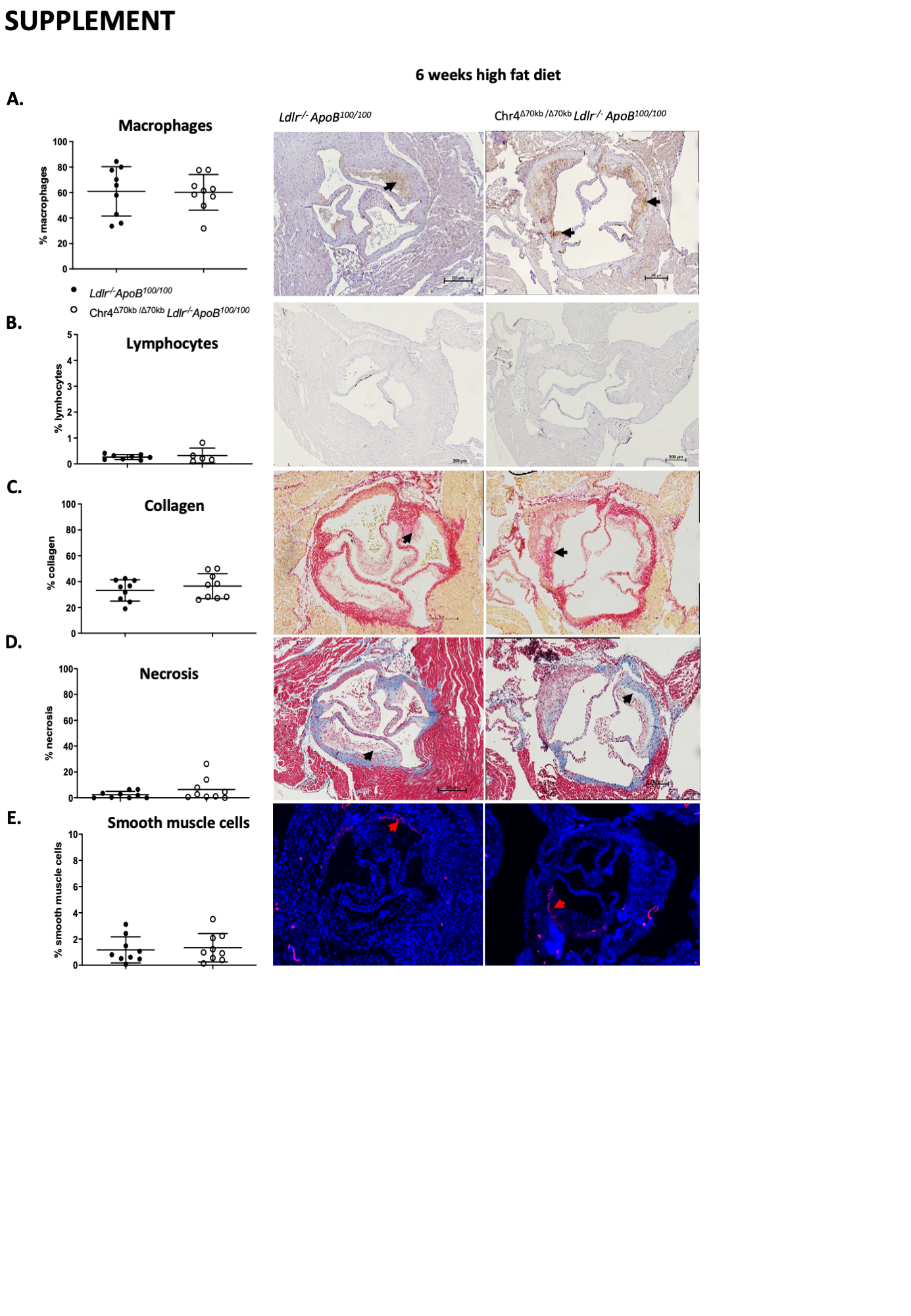


**Supplemental Figure 2. There was no difference in aortic root plaque morphology between Chr4^Δ70kb /Δ70kb^*Ldlr^-/-^ApoB^100/100^* and *Ldlr^-/-^ApoB^100/100^* mice after 6 weeks of high fat diet.**

**A)** MAC3 positive % area of Chr4^Δ70kb /Δ70kb^*Ldlr^-/-^ApoB^100/100^* (n=9) and *Ldlr^-/-^ApoB^100/100^* (n=9) mice representing macrophages, **B)** CD3e positive % area representing lymphocytes (n=8+5), **C)** Sirius red staining % representing collagen (n=9+9), **D)** Necrotic area % analysed from Masson’s Trichrome staining (n=9+9), and **E)** Fluorescent aSMA positive % area representing smooth muscle cells with DAPI counterstain for nucleus (n=9+8) in aortic root cross sections after 6 weeks high fat diet.

Graphs show mean ± SD. Statistical analyses were performed using Student t test and difference between the groups was considered statistically significant when * P ≤ 0.05.


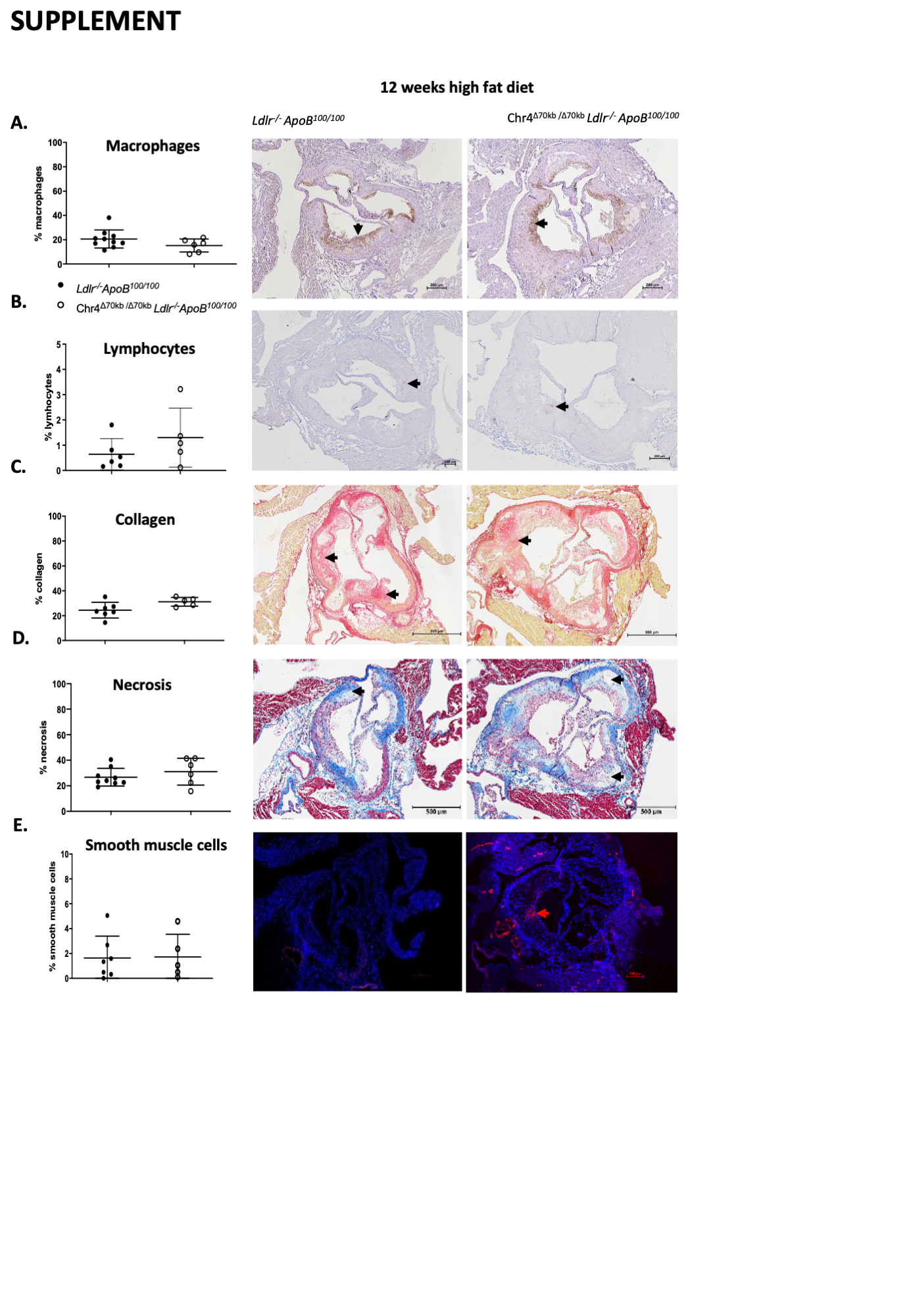


**Supplemental Figure 3. There was no difference in aortic root plaque morphology between Chr4^Δ70kb /Δ70kb^*Ldlr^-/-^ApoB^100/100^* and *Ldlr^-/-^ApoB^100/100^* mice after 12 weeks of high fat diet.**

**A)** MAC3 positive % area of Chr4^Δ70kb /Δ70kb^*Ldlr^-/-^ApoB^100/100^* (n=6) and *Ldlr^-/-^ApoB^100/100^* (n=10) mice representing macrophages, **B)** CD3e positive % area representing lymphocytes (n=6+5), **C)** Sirius red staining % representing collagen (n=5+7), **D)** Necrotic area % analysed from Masson’s Trichrome staining (n=6+9) and **E)** Fluorescent aSMA positive % area representing smooth muscle cells with DAPI counterstain for nucleus (n=5+7) in aortic root cross sections after 12 weeks high fat diet.

Graphs show mean ± SD. Statistical analyses were performed using Student t test and difference between the groups was considered statistically significant when * P ≤ 0.05.


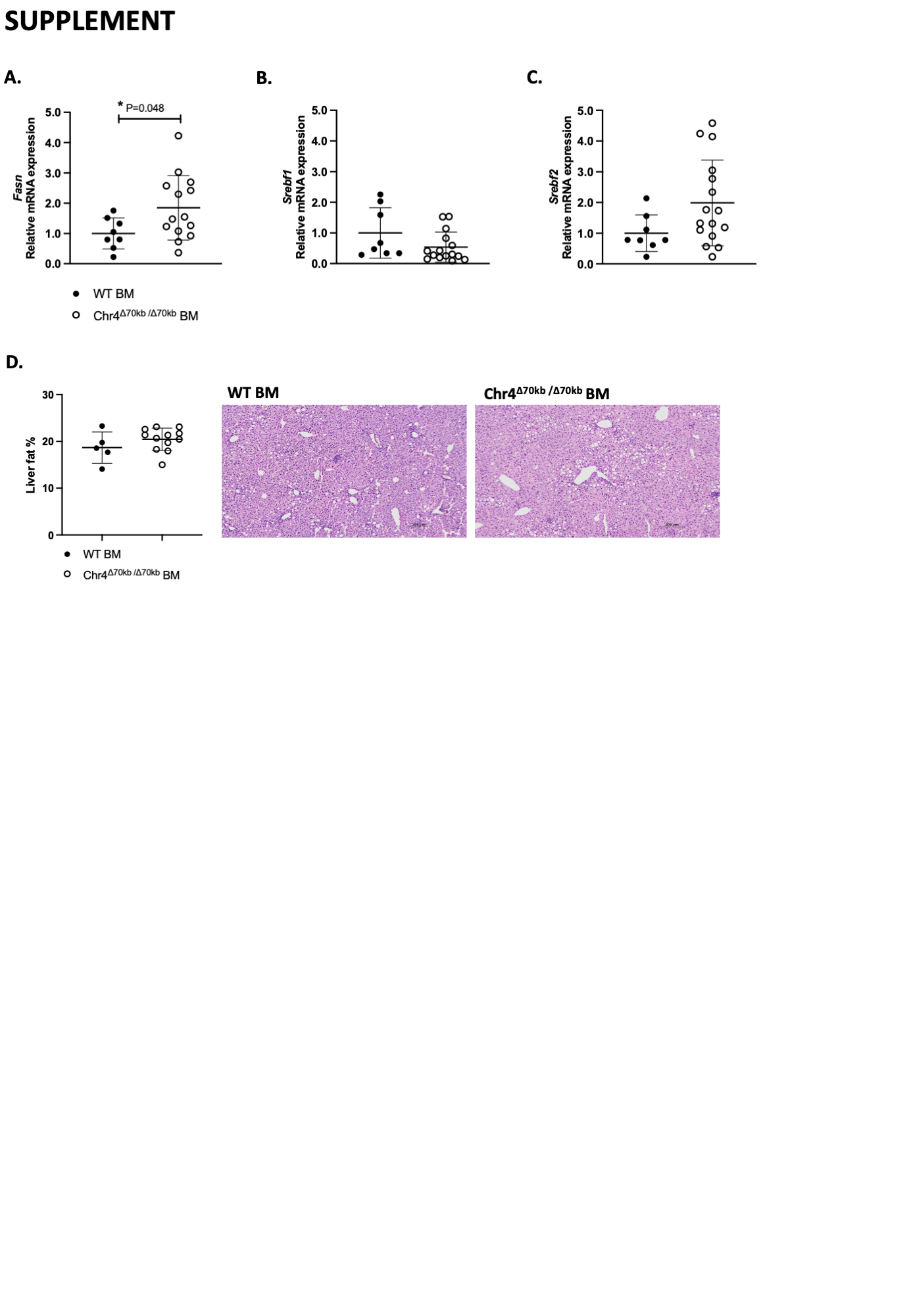


**Supplemental figure 4. Hematopoietic deletion of murine ortholog of the 9p21.3 CAD risk interval leads to increased expression of fatty acid synthesis gene *Fasn* but had no effect on *Srebf1* and -*2* in liver.**​ **The hematopoietic knockout did not affect liver lipid accumulation.**

**A-C)***Fasn*, *Srebf1* and *Srebf2* expression in Chr4^Δ70kb /Δ70kb^BM transplant and WT BM transplant recipient *Ldlr^-/-^Apo^B100/100^* mice livers after 12-week HFD. **D)** Liver lipid accumulation represented as percentage of lipid droplets in H&E-stained liver sections of Chr4^Δ70kb /Δ70kb^ (n=12) and WT BM (n=5) transplant recipient mice after 12-week HFD.

Measured mRNA levels were normalized to endogenous control *Gapdh* or *Ppia*, and analysis of relative gene expression levels were made by using 2–∆∆Ct method. Graphs show mean ± SD. Statistical analyses were performed using Student *t* test and difference between the groups was considered statistically significant when * P ≤ 0.05. ​


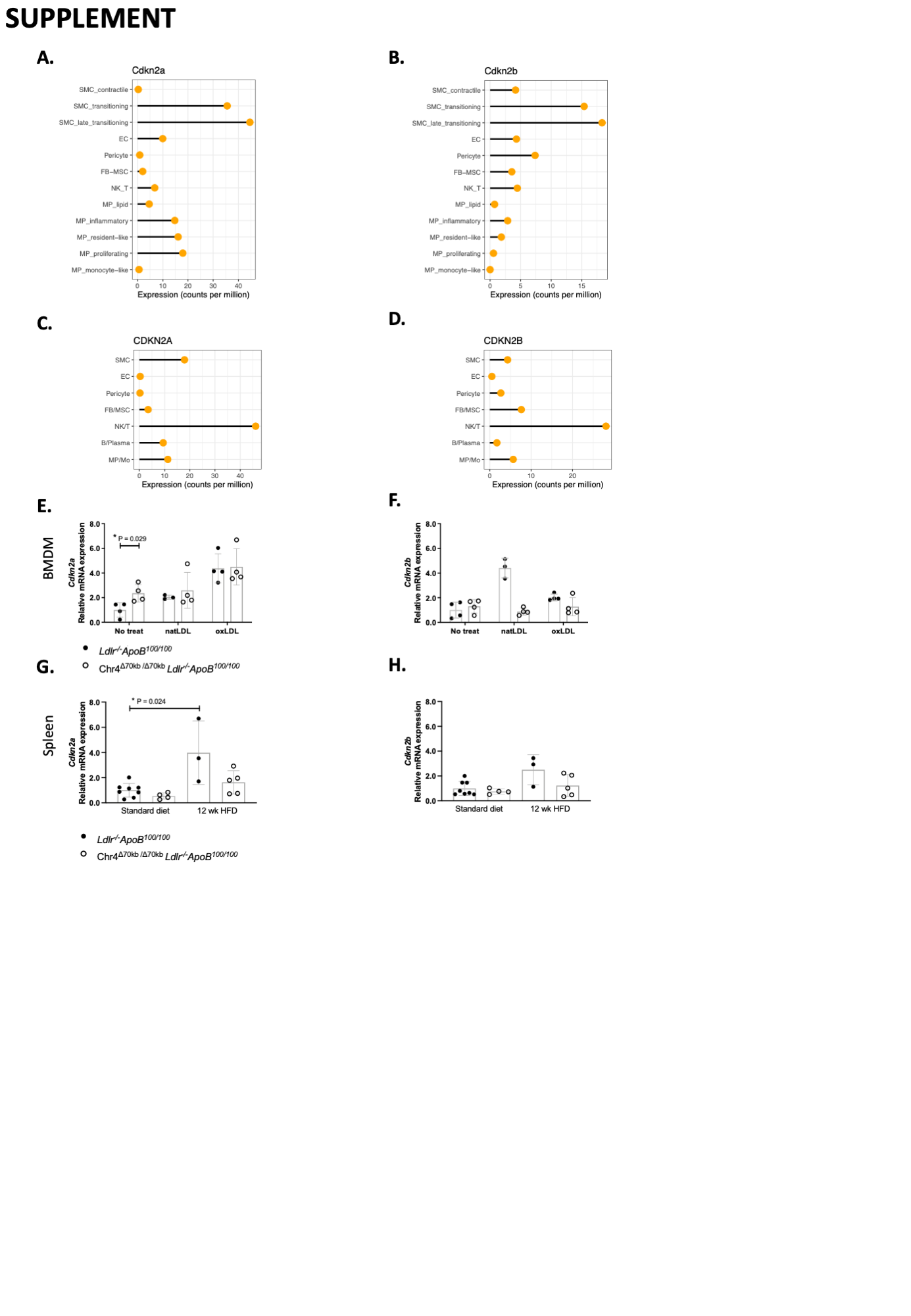


**Supplemental Figure 5. Deficiency in the murine CAD risk locus ortholog increased the basal *Cdkn2a* expression in BMDMs, but no significant differences were detected in response to LDL between the genotypes. Splenic *Cdkn2a* expression was upregulated by HFD in *Ldlr^-/-^ApoB^100/100^* mice, while in Chr4^Δ70kb/Δ70kb^*Ldlr^-/-^ApoB^100/100^*mice spleens the expression levels did not differ between the standard laboratory diet and HFD.**

**A-B)** *Cdkn2a* and *-b* expression by a cell type in mouse atherosclerotic aortas, from single cell RNA sequencing (Pan et al. 2020). **C-D)** *CDKN2A* and *-B* expression by a cell type in atherosclerotic plaques of human coronaries from single cell RNA sequencing (Wirka et al. 2019). **E-F)** Relative *Cdkn2a* and *-b* expression in Chr4^Δ70kb /Δ70kb^ *Ldlr^-/-^ApoB^100/100^* and *Ldlr^-/-^ApoB^100/100^* BMDMs after 16h native LDL & oxLDL treatment. **G-H)** Relative *Cdkn2a* & *-b* expression in Chr4^Δ70kb/Δ70kb^ *Ldlr^-/-^ApoB^100/100^* and *Ldlr^-/-^ApoB^100/100^* spleens after standard laboratory diet and 12 weeks HFD.

Measured mRNA levels were normalized to endogenous control *Gapdh* and analysis of relative gene expression levels were made by using 2–∆∆Ct method. Graphs show mean ± SD. Statistical analyses were performed using Student t test and difference between the groups was considered statistically significant when * P ≤ 0.05.

**MAJOR RESOURCES TABLES**

**Animals (in vivo studies)**

| **Species** | **Vendor or Source** | **Background Strain** | **Sex** | **Persistent ID / URL** |
| --- | --- | --- | --- | --- |
| **Parent** Mouse | In house strain, originating from Envigo | C57Bl/6JOlaHsd | f/m | https://www.envigo.com/model/c57bl-6jolahsd |

**Genetically Modified Animals**

|  | **Species** | **Vendor or Source** | **Background Strain** | **Other Information** | **Persistent ID / URL** |
| --- | --- | --- | --- | --- | --- |
| **Parent** | Mouse | MMRRCC | Chr4^Δ70kb/Δ70kb^ (129S6/SvEvTac-Del(4C4-C5)1Lap/Mmucd) |  | RRID:MMRRC_032091-UCD |
| **Parent** | Mouse | In house strain, originating from The Jackson Laboratory | *Ldlr^-/-^ApoB^100/100^* (B6;129S-Ldlrtm1Her Apobtm2Sgy/J) |  | RRID:IMSR_JAX:003000 |
| **Experimental animal** | Mouse | Created in the house | Chr4^Δ70kb/Δ70kb^ C57Bl/6JOlaHsd |  |  |
| **Experimental animal** | Mouse | Created in the house | Chr4^Δ70kb/Δ70kb^*Ldlr^-/-^ApoB^100/100^* |  |  |

**Antibodies**

| **Target antigen** | **Vendor or Source** | **Catalog #** | **Working concentration** | **Lot # (preferred but not required)** | **Persistent ID / URL** |
| --- | --- | --- | --- | --- | --- |
| MAC3 | BD Pharmingen™ | 553322 | 1:400 |  | https://www.bdbiosciences.com/en-in/products/reagents/western-blotting-and-molecular-reagents/purified-rat-anti-mouse-cd107b.553322 |
| anti-a-smooth muscle actin, C6198 | Sigma-Aldrich |  | 1:100 |  | https://www.sigmaa  ldrich.com/FI/en/product/sigma/c6198 |
| CD3e | Cell Signalling Technology | 99940 | 1:100 |  | https://www.cellsignal.com/products/primary-antibodies/cd3e-d4v8l-rabbit-mab/99940 |

**Diets**

| **Diet** | **Vendor or Source** | **Persistent ID / URL** |
| --- | --- | --- |
| TD.88137 Adjusted calories diet  (42% from fat) | Envigo | RMS-0716-US-02-PS-148 |
| Teklad Global 16% Protein Rodent Diet 2016 | Envigo | https://www.envigo.com/rodent-natural-ingredient-2016-diets |

**Arrays & Kits**

| **Product** | **Vendor or Source** | **Persistent ID / URL** |
| --- | --- | --- |
| Mouse Inflammation Antibody Array, ab133999 | Abcam | https://www.abcam.com/mouse-inflammation-antibody-array-membrane-40-targets-ab133999.html |

**Data & Code Availability**

Data is available upon request from the authors.

**Other**

| **Description** | **Source / Repository** | **Persistent ID / URL** |
| --- | --- | --- |
| qPCR probe assay: *Cdkn2a* | Integrated DNA Technologies | Mm.PT.58.43961185 |
| qPCR probe assay: *Cdkn2b* | Integrated DNA Technologies | Mm.PT.58.7138437 |
| qPCR probe assay: *Fasn* | Thermo Fisher Scientific | Mm00662319_m1 |
| qPCR probe assay: *Srebf1* | Thermo Fisher Scientific | Mm00550338_m1 |
| qPCR probe assay: *Srebf2* | Thermo Fisher Scientific | Mm01306293_m1 |
| qPCR probe assay:*Tnf* | Integrated DNA Technologies | Mm.PT.58.12575861 |
| qPCR probe assay: *Il6* | Integrated DNA Technologies | Mm.PT.58.10005566 |
| qPCR probe assay: *Arg1* | Integrated DNA Technologies | Mm.PT.58.8651372 |
| qPCR probe assay: *Fizz1* | Integrated DNA Technologies | Mm.PT.58.42723947.g |
| **Custom assays:** |  | **Sequences:** |
| Custom qPCR probe assay: *Ak148321* exon 3 | Integrated DNA Technologies | \| Primer 1 \| GCA CCT GGG TAG ATG TTC TTT \| \| --- \| --- \| \| Primer 2 \| GTG TGG TCT TCG TAG CAG AAA \| \| Probe \| AG CTT CTC AG AGC CAA ACC GTC AT \| |
| Custom qPCR probe assay: *Ak148321* exon 6 | Integrated DNA Technologies | \| Primer 1 \| GGC TAT ATA CTC ACC TCG GAA GA \| \| --- \| --- \| \| Primer 2 \| GAG GTA AAC CAG ATG CAG AAA GA \| \| Probe \| AA CCC TGC AT GTT CTC TCT CAG CC \| |
| Custom qPCR probe assay: *Ak148321* exon 9 | Integrated DNA Technologies | \| Primer 1 \| CCC TGG CAC ATC ATA AGC TAT T \| \| --- \| --- \| \| Primer 2 \| GCC ATA CAG TGT CTT CTC TTC C \| \| Probe \| TG AGT CAT CG GAA TTC TGT GGT TTG CA \| |
| Custom qPCR probe assay: *Ak148321* exon Circular 1 | Integrated DNA Technologies | \| Primer 1 \| GAC TAT CTC ACC ACT GGG ATT C \| \| --- \| --- \| \| Primer 2 \| TCT TCT TGC AAG TCC ATC CC \| \| Probe \| TGA CCT GAA TCT AAC TCC TTT GTT CAG CC \| |
| Custom qPCR probe assay: *Ak148321* exon Circular 2 | Integrated DNA Technologies | \| Primer 1 \| GAC TAT CTC ACC ACT GGG ATT C \| \| --- \| --- \| \| Primer 2 \| CCA GCC TTG GCT TTG TTA AG \| \| Probe \| AGG CTG AAC AAA GGA GTT AGA TTC AGG T \| |
| Custom qPCR probe assay: *Ak148321* exon Circular 3 | Integrated DNA Technologies | \| Primer 1 \| GAA TCA CCC GGA ACC ATT TC \| \| --- \| --- \| \| Primer 2 \| CAG GAC AGC AGG GCT ATA C \| \| Probe \| TGG CAA GTG CAT AAA CAG GGA TTC TCT \| |
| Custom qPCR probe assay: *Ak148321* exon Circular 4 | Integrated DNA Technologies | Primer 1 GTC ATC GGA ATT CTG TGG TTT G  Primer 2 GAA TCC CTG TTT CTA ATG CTG AAC  Probe TGT TAG CCA TAC AGT GTC TTC TCT TCC C |

**ARRIVE GUIDELINES**

The ARRIVE guidelines (<https://arriveguidelines.org/>) are a checklist of recommendations to improve the reporting of research involving animals. Key elements of the study design should be included below to better enable readers to scrutinize the research adequately, evaluate its methodological rigor, and reproduce the methods or findings.

**Study Design**

| **Groups** | **Sex** | **Age** (in the termination) | **Number (prior to experiment)** | **Number (after termination)** | **Littermates**  **(Yes/No)** | **Other description** |
| --- | --- | --- | --- | --- | --- | --- |
| 6 wk HFD test | F | 4 months | 9 | 9 | Yes |  |
| 6 wk HFD control | F | 4 months | 10 | 10 | Yes |  |
| 12 wk HFD test | F | 6 months | 12 | 12 | Yes |  |
| 12 wk HFD control | f  f | 6 months | 12 | 12 | Yes |  |
| Standard diet test | F | 6 months | 6 | 6 | Yes |  |
| Standard diet control | F | 6 months | 8 | 8 | Yes |  |
| BM transplant test | F | 8 months | 19 | 19 | Yes | 2 mice excluded from the data |
| BM transplant control | F | 8 months | 11 | 11 | Yes | 2 mice excluded from the data |

**Sample Size:** No *a prior* sample size calculation available. Breeding success of the animals was a determining factor for *in vivo* sample size. Primary cell yield was the limiting factor for the sample size the *in vitro*.

**Inclusion Criteria**

Hematopoietic knockout study: Success of the transplantation, characterized by the PCR results from peripheral leukocytes.

**Exclusion Criteria**

Hematopoietic knockout study: Insufficient BM transplantation. 2 mice from both groups (4 in total) were excluded by this criterion.

**Randomization**

When possible, data was collected and analysis done from the all the research animals (e.g. weight). However, due to the extensive amount of different analysis performed in this research project, along with the physiological limitations on obtainable sample size from single mouse (e.g. blood, amount of sections from aortic valve level), some samples needed to be shared for different purposes. Samples for these analyses were selected randomly.

Exact sample number (n) is always mentioned in results and figure legends.

**Blinding**

All histological, blood value and gene-expression analyses were made in blinded manner.
